# Establishment of a UPLC-MS/MS Method for Studying the Effect of Salt-Processing on Tissue Distribution of Twelve Major Bioactive Components of Qing'e Pills in Rats

**DOI:** 10.1155/2020/8832736

**Published:** 2020-09-17

**Authors:** Jingxia Hou, Shangyang Lin, Jinlan Lu, Yu Wu, Li Wu, Zhipeng Chen, Weidong Li

**Affiliations:** ^1^College of Pharmacy, Nanjing University of Chinese Medicine, Nanjing 210023, China; ^2^Engineering Center of State Ministry of Education for Standardization of Chinese Medicine Processing, Nanjing University of Chinese Medicine, Nanjing 210023, China

## Abstract

Qing'e pills is clinically used for treating osteoporosis in postmenopausal women in China. Eucommiae Cortex and Psoraleae Fructus are the main herbs of Qing'e pills and are both required to be salt-processed. In order to study the influence of salt-processing on the tissue distribution of Qing'e pills, a UPLC-MS/MS method was established for studying the tissue distribution of 12 main bioactive ingredients of Qing'e pills in rats. The linear relationships of the 12 compounds in each tissue were good. The method was fully validated for its selectivity, accuracy, precision, stability, matrix effect, and extraction recovery. Then, the validated method was successfully applied for simultaneous determination of the 12 chemical components in Qing'e pills in tissues for the first time. Areas under the curve (AUC) results showed that, except for pinoresinol diglucoside, psoralen, and isopsoralen, the distribution of the other components was increased in the kidney, uterus, ovary, and testes. Relative targeting efficiency (RTE) results showed that all 12 chemical components targeted the kidney and sexual organs. The results indicated that the Eucommiae Cortex and Psoraleae Fructus after salt-processing could significantly increase the distribution of components to the kidney and generative organs.

## 1. Introduction

Qing'e pills is included in the 2015 edition of the Chinese Pharmacopoeia, which has the effect of strengthening the kidney and waist [1]. It is clinically used for the treatment of osteoporosis in postmenopausal women. The causes of osteoporosis in postmenopausal women are mainly ascribed to estrogen deficiency, endocrine dysfunction, and metabolic disorder. Moreover, the postmenopausal osteoporosis is characterized by an imbalance in bone formation and bone resorption. Because bone resorption function is superior to bone formation, an imbalance occurs in bone remodeling. Qing'e pills consist of Eucommiae Cortex (salt-processed), Psoraleae Fructus (salt-processed), Juglandis Semen (fried), and *Allii Sativi* Bulbus [1], of which Eucommiae Cortex and Psoraleae Fructus represent the main components. Modern pharmacological studies have shown that Eucommiae Cortex increases bone density, improves trabecular microstructure, and inhibits bone mass reduction and bone strength decrease [2]. Psoraleae Fructus plays a role in hormone regulation [3] and bone strengthening [4].

Through HPLC, LC-MS/MS, and other modern instrumental analysis, it was found that the chemical components in Qing'e pills were complex, and diverse ingredients were identified mainly including chlorogenic acid, geniposidic acid, pinoresinol diglucoside, psoralen, isopsoralen, imperatorin, bergapten, trioxsalen, bavachin, neobavaisoflavone, psoralidin, isobavachalcone, bavachinin A, corylifol A, and bakuchiol [5–12]. Geniposidic acid, chlorogenic acid, and pinoresinol diglucoside are representative components of Eucommiae Cortex, belonging to iridoids, phenylpropanoids, and lignans, respectively [13]. The other 12 components are all from Psoraleae Fructus, and the main active components are coumarins, flavonoids, and monoterpenoid phenols [14, 15]. Latest literature reported that 15 kinds of main ingredients were tested in Qing'e pills by HPLC, which were geniposidic acid, pinoresinol diglucoside, psoralenoside, isopsoralenoside, psoralen, isopsoralen, isobavachin, neobavaisoflavone, bavachin, bavachalcone, psoralidin, isobavachalcone, bavachinin, corylifol A, and bakuchiol [16]. However, isopsoralenoside, corylifol A, and bakuchiol were not determined in tissues by UPLC-MS/MS.

Meridian tropism theory is one of the basic theories of traditional Chinese medicine (TCM) and plays an important role in the choice of TCM in clinical syndrome differentiation [17]. According to this theory, TCM has a special targeting effect on certain organs and meridian systems of the human body and has special therapeutic effects on diseases of these systems or organs [18, 19]. TCM processing refers to the traditional methods and techniques for processing Chinese herbal medicines into TCM samples according to the requirements of TCM. Drug processing does not only improve the drug effects and change the meridian orientation of the original drug but also reduce the toxic side effects and facilitate storage. Salt-frying is one of the TCM processing methods. The theory of meridian tropism in TCM includes the “Salt-processed drugs improving target to the kidney,” in which “kidney” refers to the kidney and sexual organs (uterus, ovary, and testes).

This paper developed a UPLC-MS/MS method for studying the tissues distribution of bioactive ingredients of Qing'e pills in rats. Twelve bioactive compounds in Eucommiae Cortex and Psoraleae Fructus were selected including pinoresinol diglucoside, geniposidic acid, psoralenoside, psoralen, isopsoralen, bavachin, isobavachin, bavachalcone, isobavachalcone, neobavaisoflavone, bavachinin, and psoralidin. The distribution of 12 bioactive ingredients in different tissues was determined by the validated UPLC-MS/MS method.

## 2. Experimental

### 2.1. Reagents and Chemicals

**Figure 1:**
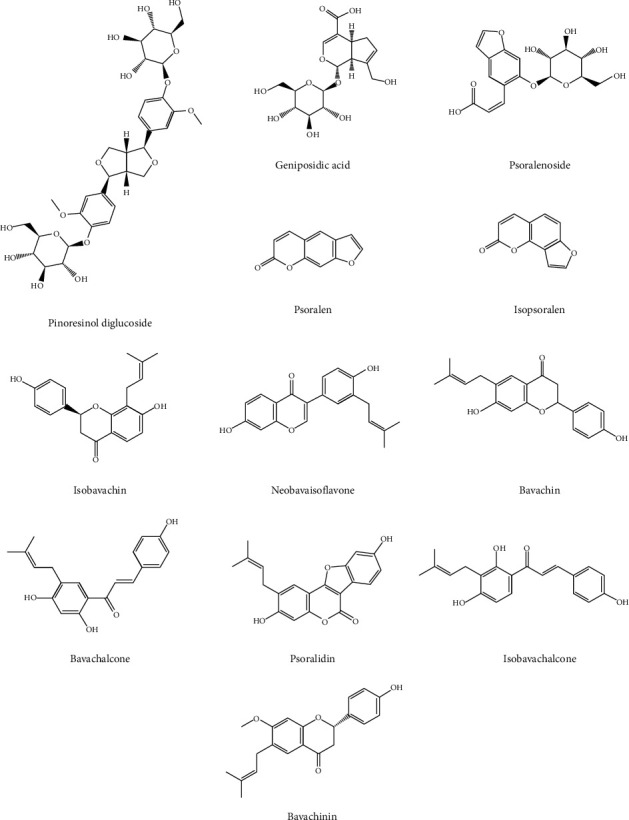
Chemical structures of 12 standard compounds.

Eucommiae Cortex and Psoraleae Fructus were purchased from Nanjing Haichang Chinese Medicinal Decoction Pieces Factory (Nanjing, China) and authenticated by Professor Jianwei Chen (Nanjing University of Chinese Medicine, Nanjing, China). Voucher specimens (number NJUTCM-20180816) were deposited at the Chinese Medicinal Herbarium of Jiangsu Key Laboratory of Chinese Medicine Processing (Nanjing, China). Standards of geniposidic acid, pinoresinol diglucoside, psoralenoside, psoralen, isopsoralen, isobavachin, neobavaisoflavone, bavachin, bavachalcone, psoralidin, isobavachalcone and bavachinin, scoparone, and rhein (internal standard (IS)) were purchased from Nanjing Shizhou Biotechnology Co., Ltd. (Nanjing, China). The purities of all the standards were above 98%. HPLC-grade methanol and acetonitrile were purchased from Calepure Company Ltd. (Canada). HPLC-grade formic acid was a product of E. Merck (Merck, Darmstadt, Germany). Ultrapure water was prepared by the Milli-Q ultrapure water purification system (Millipore Corporation, Bedford, MA, USA). All of the other relative reagents were of analytical grade. The molecular structures of the investigated compounds are shown in Figure 1.

### 2.2. UPLC-MS/MS Instruments and Analytical Conditions

The UPLC-MS/MS system consisted of a Shimadzu UPLC system, which was equipped with a LC-10 ATvp binary pump (Shimadzu Corporation UFLC XR, Kyoto, Japan) and a 5,500 triple quadrupole mass spectrometer. The latter was equipped with an electrospray ionization (ESI) source (AB SCIEX, Foster City, CA, USA). The separation of the analytes was achieved on a Waters BEH-C_18_ column (100 mm × 2.1 mm, 1.7 *μ*m). The chromatographic conditions in negative and positive ionization modes were as follows: injection volume of 2 *μ*L; column temperature at 40°C; and flow rate at 0.3 mL/min. The mobile phase was composed of 0.1% formic acid aqueous solution (A)-acetonitrile (B). The gradient elution procedures in the negative ionization mode were as follows: 0-1 min, 5% B; 1–1.5 min, 5–15% B; 1.5–2.5 min, 15–90% B; 2.5–4.5 min, 90% B; 4.5–5 min, 90–5% B; and 5–5.5 min, 5% B. The positive ionization mode elution conditions were as follows: 0-1 min, 15–34% B; 1–3 min, 34–35% B; 3–3.7 min, 35–45% B; 3.7–4.2 min, 45–50% B; 4.2–8.2 min, 50–65% B; 8.2–9.2 min, 65–85% B; 9.2–9.7 min, 85–15% B; and 9.7–10.2 min, 15% B. The optimized parameters were as follows: ion source temperature (TEM), 550°C; curtain gas (CUR), 35 psi; ion source gas 1 (GAS1), 55 psi; ion source gas 2 (GAS2), 55 psi; and ion spray voltage (IS), 5,500 V. The multiple reaction monitoring (MRM) was chosen for the quantification of the components. The precursor ions, product ions, declustering potential (DP), and collision energy (CE) for each analyte and IS in negative and positive ionization modes are shown in Table 1.

### 2.3. Preparation of Crude and Salt-Processed Qing'e Pills Extracts

Crude (500.0 g) and salt-processed (500.0 g) Qing'e pills were soaked in 4,000 mL 95% ethanol and pure water, heated, and refluxed twice for 1 h. The extracts were combined and condensed to 500 mL separately to yield crude and salt-processed Qing'e pills extracts. 1 mL of concentrated liquid was equivalent to 1 g of Qing'e pills. Final solutions were stored at 4°C before use.

### 2.4. Preparation of Calibration Standards and Quality Control Samples

The stock solutions of geniposidic acid, pinoresinol diglucoside, psoralenoside, psoralen, isopsoralen, isobavachin, neobavaisoflavone, bavachin, bavachalcone, isobavachalcone, and bavachinin were prepared by dissolving accurately the weighed reference substance in methanol at a concentration of 1 mg/mL. Psoralidin was prepared at a concentration of 0.5 mg/mL. The stock solutions were diluted into serial standard solutions. The stock solution of IS, including rhein (negative ionization mode) and scoparone (positive ionization mode), were prepared by dissolving rhein (2.5 mg) and scoparone (5.0 mg) in methanol at concentrations of 50 *μ*g/mL and 1 mg/mL, respectively. Working solutions of IS were prepared by serial dilution of the stock solutions with methanol at a concentration of 500 ng/mL for rhein and 100 ng/mL for scoparone.

Calibration samples were prepared by using 90 *μ*L of blank tissue, 10 *μ*L of standard serial solution, and 10 *μ*L of IS (50 ng/mL rhein and 100 ng/mL scoparone) to make the equivalent concentration of 1, 5, 10, 50, 250, 500, and 1,000 ng/m L of various tissue samples. Quality control (QC) solutions of the 12 compounds were prepared by adding standard solution with specified concentration into blank tissue, the samples with low, medium, and high concentrations according to the operation under “2.2.” All solutions were stored at 4°C before use.

### 2.5. Method Validation

Method validation was performed according to FDA's Guidance for Industry on Bioanalytical Method Validation [20].

#### 2.5.1. Specificity

The specificity of the method was investigated by comparing chromatograms of blank tissue homogenate samples, blank tissue homogenate samples spiked with standard solution and IS, and treated tissue homogenate samples.

#### 2.5.2. Linearity and Quantification

Various concentrations of 12 active ingredient calibration standard solutions with IS rhein (50 ng/m L, negative ionization ion mode) and scoparone (100 ng/m L, positive ionization ion mode) were added to blank tissue treated as tissue samples and assayed by using UPLC-MS/MS. The calibration curve was established via the 1/*x*^2^ weighted linear least squares regression model. LLOQ had the lowest concentrations with signal-to-noise ratio ≥10, evaluated by analyzing samples in six replicates. The lower limit of detection (LLOD) was defined as the amount that could be detected with a signal-to-noise ratio ≥3.

#### 2.5.3. Precision and Accuracy

Accuracy and precision of the method were determined by repeated analyses of QC and LLOQ samples. The intraday precision and accuracy of the method were assessed by determining QC samples six times within a single day, while the intraday precision and accuracy were estimated by determining QC samples over three consecutive days.

#### 2.5.4. Recovery and Matrix Effects

The extract recovery was calculated by comparing the peak areas of extracted QC samples with peak areas of 12 active ingredients reference standard solutions. Matrix effects of the method were determined by comparing peak areas of blank tissue extracts spiked with standard samples with peak areas of neat standard solution.

#### 2.5.5. Stability

The stability of analytes in tissues was evaluated by measuring three concentrations of the QC samples (*n* = 6) under different conditions. The short-term stability was investigated by exposing the QC samples at 25°C for 4 h. The long-term stability was assessed after storing the QC samples at −20°C for 30 days. Freezing-thawing stability was determined after QC samples underwent three freezing-thawing cycles by freezing at −20°C and thawing at 37°C in a waterbath.

### 2.6. Tissue Distribution Study

A total of 84 Sprague-Dawley rats, half male and half female, were randomly divided into two groups (crude and salt-processed Qing'e pills groups). The rats were fed for a week and fasted for 12 h before the experiment. This experiment has been approved by the Animal Ethics Committee of Nanjing University of Chinese Medicine, license Number: 201903A011. These two groups of rats were orally administered raw and salt-processed extracts at the same dose of 1.2 mL/200 g of body weight. The rats were sacrificed at 10, 30, 90, 180, 360, 480, and 720 min (for each time point, 6 rats were sacrificed for each group, half male and half female). The heart, liver, spleen, lung, kidney, ovary, uterus, and testicular tissue samples were collected from the rats, the surface blood was washed with 0.9% normal saline solution, and the tissues were dried with filter paper. Different organ tissues were weighed, and two volumes of iced normal saline solution were added to obtain the homogenates, which were stored at −20°C for further analysis.

Each tissue homogenate (90 *μ*L) was placed in a 1.5 mL centrifuge tube, and 10 *μ*L of the internal standard solution was added. Acetonitrile (300 *μ*L) was added, vortexed for 5 min, and centrifuged at 11308.75 ×g for 5 min. The supernatants were transferred into the 1.5 mL Eppendorf tube, and after centrifugal concentration, 100 *μ*L methanol was added and centrifuged at 11308.75 ×g for 5 min before being vortexed for 5 min. Finally, 80 *μ*L of the supernatants was used for UPLC-MS/MS.

### 2.7. Targeting Efficiency Evaluation

The purpose of salt-processing of Qing'e pills in TCM theory is somewhat similar to modern drug target-delivery theory. AUC and RTE were utilized to investigate the effect of salt-processing on tissue distribution of Qing'e pills compounds. The relevant parameters were calculated according to previously described equations [21, 22]:

(1) $$\begin{array}{c} \text{RTE}=\frac{\left({\text{AUC}}_{\text{salt}-\text{processed}}/{\text{AUC}}_{\text{sum}}\right)-\left({\text{AUC}}_{\text{crude}}/{\text{AUC}}_{\text{sum}}\right)}{{\text{AUC}}_{\text{crude}}/{\text{AUC}}_{\text{sum}}},\\ {\text{AUC}}_{\text{sum}}={\text{AUC}}_{\text{heart}}+{\text{AUC}}_{\text{liver}}+{\text{AUC}}_{\text{spleen}}+{\text{AUC}}_{\text{lung}}+{\text{AUC}}_{\text{kidney}}+{\text{AUC}}_{\text{ovary}\left(\text{testis}\right)}+{\text{AUC}}_{\text{uterus}\left(\text{male rat is }0\right)}. \end{array}$$

In these equations, AUC_sum_ involves the sum of AUC of all tissues in salt-processed and crude groups, respectively.

### 2.8. Data Analysis

Data analysis was performed using DAS 2.0 software and SPSS 16.0 software. AUC was obtained using the DAS 2.0 software noncompartment model by the obtained concentration of each time point component. The AUC salt group data in the data analysis were subjected to composition conversion and compared to the raw product. The calculation formula is as the following equation:

(2) $$\begin{array}{c} {\text{AUC}}_{\text{salt}-\text{processed}}\text{group}=\frac{{\text{AUC}}_{\text{salt}-\text{processed}}\text{group}}{\text{contents of salt}-\text{processed}/\text{contents of crude}}. \end{array}$$

## 3. Results and Discussion

### 3.1. Method Optimization of UHPLC-MS/MS Conditions

#### 3.1.1. Specificity

**Figure 2:**
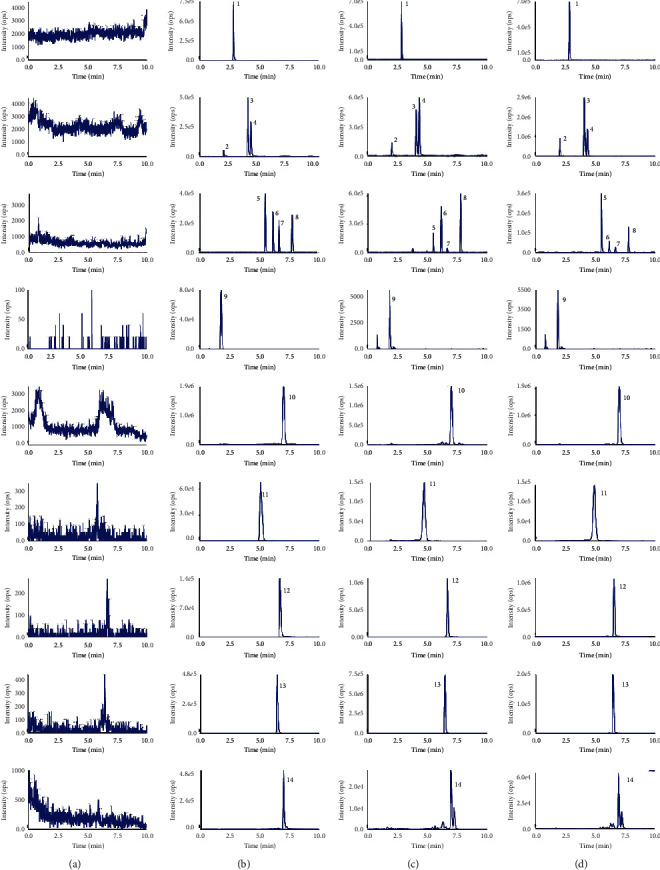
SIM chromatograms of 12 active ingredients and IS in rat tissue. (a) Blank tissue; (b) blank tissue spiked with standard solution and IS; (c) kidney tissue samples after intragastric administration of crude Qing'e pills; and (d) kidney tissue samples of oral administration of salt-processed Qing'e pills (1. Escoparone, 2. psoralenoside, 3. psoralen, 4. Isopsoralen, 5. isobavachin, 6. bavachin, 7. bavachalcone, 8. isobavachalcone, 9. pinoresinol diglucoside, 10. rhein, 11. geniposidic acid, 12. psoralidin, 13. neobavaisoflavone, and 14. bavachinin).

As shown in Figure 2, the peak shapes measured under experimental conditions did not affect each other, and the endogenous substances in the heart, liver, spleen, lung, kidney, ovary, uterus, and testes did not interfere with the peaks of the detected components in each group.

#### 3.1.2. Linearity and Sensitivity

Under UPLC-MS/MS conditions, the equations for the calibration curves, correlation coefficient, linear range, and lower limit of quantification (LLOQ) of 12 bioactive ingredients are shown in Table 2. The results showed that the linear relationships of the 12 compounds in the linear range of the methanol solution and each tissue were good, and the ranges of concentrations and limits were suitable for the determination of Qing'e pills extract content and tissue distribution.

#### 3.1.3. Precision and Accuracy

The precision and accuracy of the assay were evaluated using QC samples at low, medium, and high concentrations. The results for kidneys are shown in Table 3. The accuracy and precision of the method were within the specified range, meeting the relevant requirements for biological sample determination.

#### 3.1.4. Extract Recovery and Matrix Effect

The extract recoveries and matrix effects in kidney are presented in Table 4. The extract recoveries of QC samples at low, medium, and high concentrations were 81.59–90.84%, while the matrix effects of QC samples were 90.85–96.38%. The RSD of extract recoveries was less than 7.28%, and the matrix effects were less than 9.39%. The results indicated that the method was suitable for the treatment of kidney tissue samples in this experiment.

#### 3.1.5. Stability

The results of stability in the kidney tissue under different storage conditions are presented in Table 5. The samples were all stable, and the storage conditions did not affect the determination of the chemical components in the experimental tissue samples.

### 3.2. Tissue Distribution Studies

**Figure 3:**
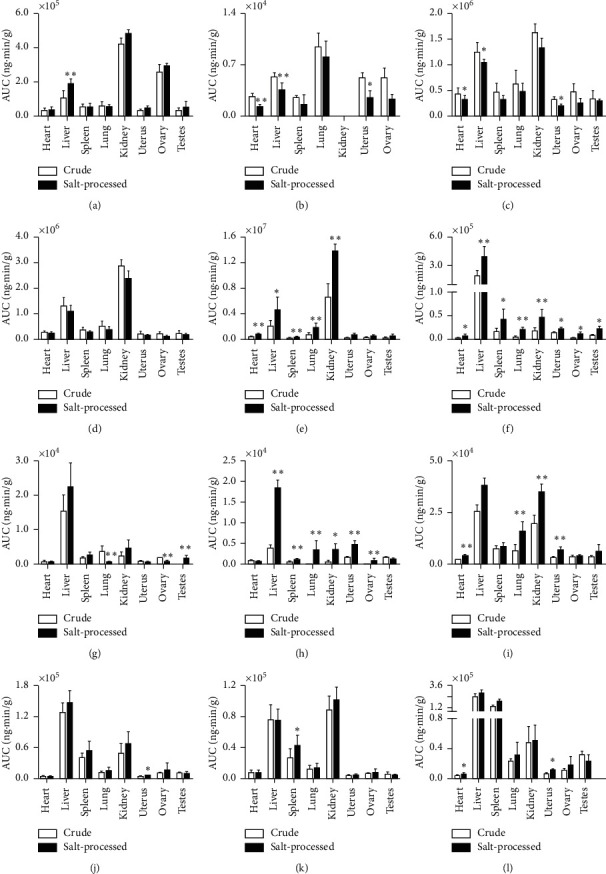
The AUC profiles of 12 kinds of compounds in crude and salt-processed Qing'e pills in rat tissues (*n* = 3 for sexual organs and *n* = 6 for other tissues). The salt-processed group compared to the crude group, ^*∗*^*p* < 0.05; ^*∗∗*^*p* < 0.01. (a) Geniposidic acid, (b) pinoresinol diglucoside, (c) psoralen, (d) isopsoralen, (e) psoralenoside, (f) bavachin, (g) isobavachin, (h) bavachalcone, (i) isobavachalcone, (j) neobavaisoflavone, (k) psoralidin, and (l) bavachinin.

The AUC profiles of 12 compounds in crude and salt-processed Qing'e pills are shown in Figure 3. The psoralenoside, bavachin, isobavachalcone, and neobavaisoflavone were mainly distributed in the kidney and liver. After salt-processing, the distribution of psoralenoside, bavachin, isobavachalcone, and neobavaisoflavone increased in the kidney, uterus, ovary, and testes (*p* < 0.05). Bavachalcone was mainly distributed in the liver, uterus, and testes, and the distribution in the uterus, kidney, and ovary increased after salt-processing (*p* < 0.05). The crude bavachinin and isobavachin were mainly distributed in the liver, kidney, spleen, and lung. After salt-processing, distribution of them increased in the uterus, testes, and heart (*p* < 0.05). Geniposidic acid and psoralidin were mainly distributed in the kidney, ovary, and liver. After salt-processing, distribution of them in the liver and spleen increased (*p* < 0.05). Pinoresinol diglucoside and psoralen were mainly distributed in the kidney, lung, liver, uterus, and ovary before salt-processing. Compared to crude products, the distribution of them were decreased after salt-processing. Isopsoralen was mainly distributed in the kidney and liver tissues, and there was no significant change in the distribution after salt-processing (*p* > 0.05).

**Figure 4:**
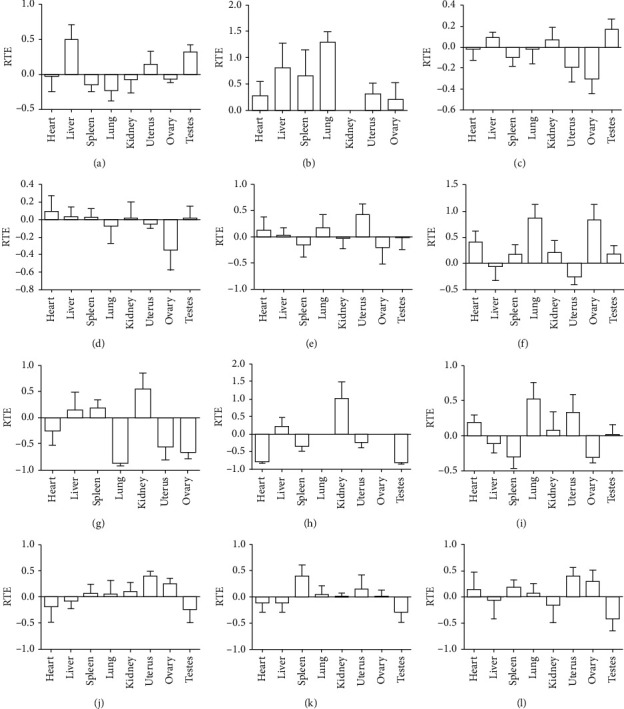
The RTE profiles of 12 kinds of compounds in crude and salt-processed Qing'e pills in rat tissue (*n* = 3 for sexual organs and *n* = 6 for other tissues). (a) Geniposidic acid, (b) pinoresinol diglucoside, (c) psoralen, (d) isopsoralen, (e) psoralenoside, (f) bavachin, (g) isobavachin, (h) bavachalcone, (i) isobavachalcone, (j) neobavaisoflavone, (k) psoralidin, and (l) bavachinin.

RTE indicates the proportion of drug distribution of the salt-processed product relative to the crude product. RTE > 0 indicates that the component of the salt-processed product enters the tissue. The larger the RTE, the more effective the targeting effect. The RTE of 12 components is shown in Figure 4. The target of geniposidic acid was the liver (0.50), testes (0.32), and uterus (0.14). The target of pinoresinol diglucoside was the lung (1.30), liver (0.81), spleen (0.66), uterus (0.31), heart (0.27), and ovary (0.20). The target of psoralen was the testes (0.17), liver (0.09), and kidney (0.07). The target of isopsoralen was the heart (0.09), liver (0.03), spleen (0.03), kidney (0.02), and testes (0.02). The target of psoralenoside was the uterus (0.43), lung (0.18), heart (0.13), and liver (0.02). The target of bavachin was the lung (0.86), ovary (0.82), heart (0.40), kidney (0.20), testes (0.17), and spleen (0.16). The target of isobavachin was the testes, kidney (0.55), spleen (0.18), and liver (0.14). The target of bavachalcone was the ovary, lung, kidney (1.02), and liver (0.21). The target of isobavachalcone was the lung (0.52), uterus (0.33), heart (0.19), kidney (0.07), and testes (0.01). The target of neobavaisoflavone was the uterus (0.40), ovary (0.25), kidney (0.10), spleen (0.07), and lung (0.05). The target of psoralidin was the spleen (0.39), uterus (0.14), lung (0.04), kidney (0.002), and ovary (0.006). The target of bavachinin was the uterus (0.40), ovary (0.30), spleen (0.19), heart (0.15), and lung (0.08).

### 3.3. Discussion

Except for pinoresinol diglucoside, psoralen, and isopsoralen, the tissue distribution of other nine ingredients were increased after salt-processing in general. The distribution of psoralenoside, bavachin, isobavachin, bavachalcone, isobavachalcone, neobavaisoflavone, and bavachinin into the kidney increased. The distribution of geniposidic acid in the liver increased after salt-processing. The distribution of psoralidin into the spleen increased after salt-processing. Since the difference between the salt-processed group and the crude group was deducted from the in vitro content determination, the increase in the distribution of these nine components in the organs after salt-processing was not related to the concentration difference. The increased distribution of most components in the organs of the salt-processed group may be related to the high osmotic pressure caused by salt [23]. The decreased distribution of pinoresinol diglucoside and psoralen in organs may be due to the transformation of salt and other chemicals in vivo.

RTE showed that all twelve chemical components targeted the kidney or sexual organs (uterus, ovary, and testes) to some extent. After salt-processing, the components targeting the kidney were psoralen, isopsoralen, bavachin, isobavachin, bavachalcone, isobavachalcone, neobavaisoflavone, and psoralidin. The components targeting the uterus, ovary, and testes were geniposidic acid, pinoresinol diglucoside, psoralen, isopsoralen, psoralenoside, bavachin, isobavachin, bavachalcone, isobavachalcone, neobavaisoflavone, psoralidin, and bavachinin. Bavachalcone, isobavachin, bavachinin, psoralenoside, neobavaisoflavone, and psoralen targeted the kidney and sexual organs the most.

The results of AUC and RTE indicated that salt-processing in Qing'e pills not only can enhance the dissolution of the effective ingredients but also increase the targeting of drugs into the kidney and sexual organs, which may be the potential reason for Qing'e pills to play the role of tonifying the liver and kidney and strengthening muscles and bones.

One of the main functions of TCM salt-processing was to tonify the kidney, and from the perspective of TCM syndrome differentiation and treatment, the main bone of the kidney gave birth to marrow, which was the innate basis, so salt-processing plays an important role in the treatment of osteoporosis [24, 25]. The bioactive ingredients in Qing'e pills have certain pharmacological activities. Bavachalcone can inhibit osteoclastogenesis by interfering with the ERK and Akt signaling pathways and the induction of c-Fos and NFATc1 during differentiation [26]. Isobavachin, bavachinin, psoralenoside, neobavaisoflavone, and psoralen may be associated with isopentenyl and estrogen-like activity in the treatment of osteoporosis [3, 27–29]. Pinoresinol diglucoside, bavachin, and isobavachalcone targeted the lung. Geniposidic acid, pinoresinol diglucoside, isobavachin, and bavachalcone targeted the liver. The results indicated that salt-processing could strengthen the estrogen-like activity of salt. This enhances Qing'e pills's antiosteoporosis effect.

## 4. Conclusion

A sensitive and accurate UPLC-MS/MS for simultaneous determination of 12 bioactive components of Qing'e pills in different tissues of rats. This study investigated the effects of salt-processing on the tissue distribution of 12 main bioactive ingredients. After salt-processing, the tissue distribution of bavachalcone, isobavachin, bavachinin, psoralenoside, neobavaisoflavone, and psoralen were increased in the kidney and sexual organs. The result provided a basis for clinical usage of Qing'e pills.

## Figures and Tables

**Table 1:** The precursor ions, product ions, declustering potential (DP), and collision energy (CE) for each analyte and internal standard (IS) in negative and positive ionization modes.

Compounds	Molecular weight	Ionization mode	Monitoring ion	Parent ion	Daughter ion	DP (V)	CE (V)
Rhein (IS)	284.22	ESI^−^	[M − H]^−^	283.2	238.9	−59.15	−19.01
Geniposidic acid	374.34	ESI^−^	[M − H]^−^	373.0	211.3	−126.4	−18.65
Psoralidin	336.34	ESI^−^	[M − H]^−^	335.0	280.0	−127.91	−35.4
Neobavaisoflavone	322.37	ESI^−^	[M − H]^−^	321.0	264.8	−113.91	−38.92
Bavachinin	338.40	ESI^−^	[M − H]^−^	337.1	119.0	−59.91	−27.28
Escoparone (IS)	206.19	ESI^+^	[M + H]^+^	207.2	151.1	93.08	27.23
Psoralenoside	366.32	ESI^+^	[M + H]^+^	187.2	131.0	82.75	33.1
Psoralen	186.16	ESI^+^	[M + H]^+^	187.2	131.0	82.75	33.1
Isopsoralen	186.16	ESI^+^	[M + H]^+^	187.2	131.0	82.75	33.1
Bavachin	324.37	ESI^+^	[M + H]^+^	325.4	149.0	79.14	30.73
Isobavachin	324.37	ESI^+^	[M + H]^+^	325.4	149.0	79.14	30.73
Bavachalcone	324.37	ESI^+^	[M + H]^+^	325.4	149.0	79.14	30.73
Isobavachalcone	324.37	ESI^+^	[M + H]^+^	325.4	149.0	79.14	30.73
Pinoresinol diglucoside	682.67	ESI^+^	[M + H_2_O]^+^	700.5	234.9	86.44	19.14

**Table 2:** Calibration curves, correlation coefficient, linear range, and LLOQ of 12 compounds.

Compound	Tissue	Linear regression equation	Correlation coefficient (*r*)	Linear range (ng/mL)	LLOQ (ng/mL)
Psoralen	Heart	*Y* = 7.77 × 10^−3^*X* + 2.23 × 10^−2^	1.0000	1–1000	1.0
	Liver	*Y* = 3.60 × 10^−3^*X* + 1.03 × 10^−1^	1.0000	5–1000	5.0
	Spleen	*Y* = 5.71 × 10^−3^*X* + 2.92 × 10^−2^	0.9995	5–1000	5.0
	Lung	*Y* = 2.44 × 10^−3^*X* + 3.33 × 10^−2^	0.9980	5–1000	5.0
	Kidney	*Y* = 3.44 × 10^−3^*X* + 1.21 × 10^−1^	0.9996	2–1000	2.0
	Uterus	*Y* = 8.62 × 10^−3^*X* + 8.86 × 10^−3^	1.0000	1–1000	1.0
	Ovary	*Y* = 6.53 × 10^−3^*X* + 1.79 × 10^−2^	1.0000	5–1000	5.0
	Testes	*Y* = 5.04 × 10^−3^*X* + 4.89 × 10^−2^	1.0000	1–1000	1.0

Isopsoralen	Heart	*Y* = 8.57 × 10^−3^*X* + 3.61 × 10^−2^	1.0000	1–1000	1.0
	Liver	*Y* = 1.65 × 10^−3^*X* + 1.74 × 10^−1^	0.9986	5–1000	5.0
	Spleen	*Y* = 4.55 × 10^−3^*X* + 3.50 × 10^−2^	0.9981	5–1000	5.0
	Lung	*Y* = 1.48 × 10^−3^*X* + 5.62 × 10^−2^	0.9970	5–1000	5.0
	Kidney	*Y* = 2.34 × 10^−3^*X* + 1.77 × 10^−1^	0.9994	2–1000	2.0
	Uterus	*Y* = 6.23 × 10^−3^*X* + 2.86 × 10^−2^	1.0000	1–500	1.0
	Ovary	*Y* = 6.12 × 10^−3^*X* + 5.19 × 10^−2^	1.0000	5–1000	5.0
	Testes	*Y* = 4.27 × 10^−3^*X* + 7.24 × 10^−2^	0.9997	5–1000	5.0

Bavachin	Heart	*Y* = 2.31 × 10^−2^*X* − 6.13 × 10^−3^	1.0000	0.4–50	0.4
	Liver	*Y* = 3.17 × 10^−2^*X* + 8.99 × 10^−2^	0.9977	1–500	1.0
	Spleen	*Y* = 2.61 × 10^−2^*X* + 3.94 × 10^−2^	1.0000	1–200	1.0
	Lung	*Y* = 4.07 × 10^−2^*X* + 2.90 × 10^−2^	0.9976	0.5–200	0.5
	Kidney	*Y* = 2.19 × 10^−2^*X* + 6.66 × 10^−3^	0.9999	1–500	1.0
	Uterus	*Y* = 2.11 × 10^−2^*X* − 4.30 × 10^−2^	0.9997	1–200	1.0
	Ovary	*Y* = 3.70 × 10^−2^*X* − 1.14 × 10^−2^	1.0000	1–50	1.0
	Testes	*Y* = 2.14 × 10^−2^*X* − 2.71 × 10^−2^	0.9897	1–50	1.0

Isobavachin	Heart	*Y* = 4.07 × 10^−2^*X* + 8.55 × 10^−3^	0.9996	0.3–50	0.3
	Liver	*Y* = 5.35 × 10^−2^*X* + 5.28 × 10^−2^	0.9999	1–250	1.0
	Spleen	*Y* = 3.82 × 10^−2^*X* + 8.21 × 10^−2^	0.9995	0.5–100	0.5
	Lung	*Y* = 5.94 × 10^−2^*X* + 3.32 × 10^−2^	0.9976	0.5–100	0.5
	Kidney	*Y* = 4.62 × 10^−2^*X* + 1.85 × 10^−2^	1.0000	0.1–250	0.1
	Uterus	*Y* = 3.93 × 10^−2^*X* + 3.14 × 10^−3^	1.0000	0.1–50	0.1
	Ovary	*Y* = 7.17 × 10^−2^*X* − 7.18 × 10^−2^	0.9999	1–50	1.0
	Testes	*Y* = 3.94 × 10^−2^*X* + 7.50 × 10^−2^	0.9887	0.5–50	0.5

Pinoresinol diglucoside	Heart	*Y* = 6.50 × 10^−4^*X* − 2.98 × 10^−4^	1.0000	0.5–100	0.5
	Liver	*Y* = 8.57 × 10^−4^*X* + 6.87 × 10^−5^	0.9999	0.5–250	0.5
	Spleen	*Y* = 4.46 × 10^−4^*X* + 5.35 × 10^−4^	1.0000	0.5–100	0.5
	Lung	*Y* = 6.95 × 10^−4^*X* + 1.26 × 10^−3^	1.0000	0.5–500	0.5
	Kidney	*Y* = 3.45 × 10^−4^*X* + 5.16 × 10^−4^	0.9998	1–1000	1.0
	Uterus	*Y* = 6.24 × 10^−4^*X* + 6.35 × 10^−4^	0.9996	0.5–100	0.5
	Ovary	*Y* = 7.81 × 10^−4^*X* − 3.66 × 10^−4^	0.9997	0.5–100	0.5
	Testes	*Y* = 6.90 × 10^−4^*X* + 6.48 × 10^−4^	0.9999	0.5–50	0.5

Psoralenoside	Heart	*Y* = 6.99 × 10^−4^*X* + 2.09 × 10^−3^	0.9993	5–1000	5.0
	Liver	*Y* = 4.63 × 10^−4^*X* + 1.21 × 10^−2^	0.9996	5–1000	5.0
	Spleen	*Y* = 5.56 × 10^−4^*X* + 2.73 × 10^−3^	0.9975	5–1000	5.0
	Lung	*Y* = 9.26 × 10^−4^*X* + 2.96 × 10^−2^	0.9998	1–1000	1.0
	Kidney	*Y* = 3.61 × 10^−4^*X* + 8.94 × 10^−3^	0.9991	5–2000	5.0
	Uterus	*Y* = 9.86 × 10^−4^*X* + 3.82 × 10^−2^	0.9994	5–2000	5.0
	Ovary	*Y* = 6.00 × 10^−4^*X* + 1.93 × 10^−2^	0.9998	5–2000	5.0
	Testes	*Y* = 5.16 × 10^−4^*X* + 1.57 × 10^−2^	0.9973	5–1000	5.0

Isobavachalcone	Heart	*Y* = 4.89 × 10^−2^*X* − 5.05 × 10^−2^	0.9997	1–50	1.0
	Liver	*Y* = 8.17 × 10^−2^*X* + 9.05 × 10^−2^	0.9973	1–100	1.0
	Spleen	*Y* = 4.86 × 10^−2^*X* + 6.15 × 10^−2^	0.9998	0.5–250	0.5
	Lung	*Y* = 4.93 × 10^−2^*X* + 1.59 × 10^−3^	0.9998	0.5–250	0.5
	Kidney	*Y* = 3.18 × 10^−2^*X* + 2.75 × 10^−2^	0.9993	1–500	1.0
	Uterus	*Y* = 3.47 × 10^−2^*X* − 4.89 × 10^−2^	0.9995	1–50	1.0
	Ovary	*Y* = 2.29 × 10^−2^*X* + 1.08 × 10^−2^	0.9999	0.5–50	0.5
	Testes	*Y* = 2.88 × 10^−2^*X* + 2.11 × 10^−2^	1.0000	0.5–50	0.5

Bavachalcone	Heart	*Y* = 2.47 × 10^−2^*X* − 8.09 × 10^−3^	0.9967	0.3–25	0.3
	Liver	*Y* = 2.83 × 10^−2^*X* + 1.88 × 10^−2^	0.9999	0.5–250	0.5
	Spleen	*Y* = 2.83 × 10^−2^*X* + 2.61 × 10^−2^	0.9996	0.5–50	0.5
	Lung	*Y* = 3.05 × 10^−2^*X* + 3.49 × 10^−2^	0.9997	0.5–100	0.5
	Kidney	*Y* = 3.01 × 10^−2^*X* + 2.78 × 10^−2^	0.9992	0.5–100	0.5
	Uterus	*Y* = 3.25 × 10^−2^*X* − 2.93 × 10^−2^	0.9998	0.5–250	0.5
	Ovary	*Y* = 2.17 × 10^−2^*X* + 1.12 × 10^−2^	1.0000	0.5–50	0.5
	Testes	*Y* = 2.63 × 10^−2^*X* − 1.85 × 10^−2^	0.9996	0.5–50	0.5

Geniposidic acid	Heart	*Y* = 1.11 × 10^−3^*X* + 4.13 × 10^−4^	0.9996	1–500	1.0
	Liver	*Y* = 6.88 × 10^−4^*X* + 3.58 × 10^−3^	0.9993	1–1000	1.0
	Spleen	*Y* = 1.31 × 10^−3^*X* + 1.03 × 10^−3^	0.9990	1–500	1.0
	Lung	*Y* = 8.89 × 10^−4^*X* + 1.78 × 10^−3^	0.9986	1–1000	1.0
	Kidney	*Y* = 8.11 × 10^−4^ X - 1.72 × 10^−3^	0.9992	1–2000	1.0
	Uterus	*Y* = 5.08 × 10^−3^*X* + 1.48 × 10^−2^	0.9995	0.5–500	0.5
	Ovary	*Y* = 8.04 × 10^−4^*X* + 7.50 × 10^−4^	0.9990	1–1000	1.0
	Testes	*Y* = 5.83 × 10^−4^*X* + 3.28 × 10^−4^	0.9956	1–250	1.0

Psoralidin	Heart	*Y* = 9.87 × 10^−3^*X* − 2.03 × 10^−3^	0.9999	1–250	1.0
	Liver	*Y* = 7.80 × 10^−3^*X* + 1.11 × 10^−2^	0.9997	1–500	1.0
	Spleen	*Y* = 2.43 × 10^−2^*X* − 2.60 × 10^−3^	0.9998	1–500	1.0
	Lung	*Y* = 1.54 × 10^−2^*X* + 1.70 × 10^−2^	0.9993	1–500	1.0
	Kidney	*Y* = 6.92 × 10^−2^*X* + 3.91 × 10^−3^	0.9996	1–1000	1.0
	Uterus	*Y* = 3.96 × 10^−2^*X* − 6.48 × 10^−2^	0.9996	1–250	1.0
	Ovary	*Y* = 2.11 × 10^−2^*X* + 1.84 × 10^−2^	0.9999	0.5–100	0.5
	Testes	*Y* = 1.56 × 10^−2^*X* + 1.59 × 10^−2^	0.9996	0.5–50	0.5

Neobavaisoflavone	Heart	*Y* = 1.39 × 10^−2^*X* − 5.15 × 10^−3^	0.9994	0.5–50	0.5
	Liver	*Y* = 7.88 × 10^−3^*X* + 5.31 × 10^−2^	0.9991	1–1000	1.0
	Spleen	*Y* = 2.06 × 10^−2^*X* + 4.61 × 10^−2^	0.9998	1–500	1.0
	Lung	*Y* = 1.43 × 10^−2^*X* + 3.16 × 10^−2^	1.0000	1–300	1.0
	Kidney	*Y* = 1.26 × 10^−2^*X* − 4.05 × 10^−3^	0.9994	1–1000	1.0
	Uterus	*Y* = 6.00 × 10^−2^*X* − 1.22 × 10^−1^	0.9997	1–50	1.0
	Ovary	*Y* = 1.81 × 10^−2^*X* + 1.16 × 10^−2^	0.9996	1–100	1.0
	Testes	*Y* = 1.40 × 10^−2^*X* + 1.36 × 10^−2^	0.9998	1–50	1.0

Bavachinin	Heart	*Y* = 2.72 × 10^−3^*X* − 2.72 × 10^−3^	0.9999	1–100	1.0
	Liver	*Y* = 1.48 × 10^−3^*X* + 3.93 × 10^−3^	0.9998	1–1000	1.0
	Spleen	*Y* = 7.85 × 10^−3^*X* − 2.00 × 10^−2^	0.9995	1–1000	1.0
	Lung	*Y* = 4.39 × 10^−3^*X* − 7.75 × 10^−4^	0.9994	1–500	1.0
	Kidney	*Y* = 3.51 × 10^−3^*X* − 3.14 × 10^−3^	0.9992	1–500	1.0
	Uterus	*Y* = 1.21 × 10^−2^*X* − 1.96 × 10^−2^	0.9997	1–50	1.0
	Ovary	*Y* = 6.56 × 10^−3^*X* − 9.65 × 10^−4^	0.9997	1–50	1.0
	Testes	*Y* = 2.72 × 10^−3^*X* − 2.72 × 10^−3^	0.9999	1–100	1.0

**Table 3:** Intraassay and interassay precision and the accuracy of 12 kinds of compounds in rat kidney (*n* = 6).

Compound	Concentrations (ng/mL)	Intra-day			Inter-day		
		Measured (ng/mL)	Precision (%)	Accuracy (RE%)	Measured (ng/mL)	Precision (%)	Accuracy (RE%)
Psoralen	5	4.70 ± 0.26	5.46	−6.04	4.86 ± 0.26	5.45	−2.83
	500	490.30 ± 17.05	3.48	−1.94	492.03 ± 14.95	3.04	−1.59
	800	792.43 ± 11.80	1.49	−0.95	787.82 ± 19.49	2.47	−1.52

Isopsoralen	5	4.75 ± 0.25	5.22	−5.01	4.84 ± 0.25	5.25	−3.14
	500	493.60 ± 20.35	4.12	−1.28	492.37 ± 16.57	3.37	−1.53
	800	782.55 ± 18.84	2.41	−2.18	782.18 ± 14.21	1.82	−2.23

Psoralenoside	12.50	12.37 ± 0.16	1.33	−1.05	12.40 ± 0.13	1.02	−0.78
	1000	956.47 ± 40.02	4.18	−4.35	958.53 ± 37.30	3.89	−4.15
	1600	1565.77 ± 40.30	2.57	−2.14	1551.71 ± 40.97	2.64	−3.02

Bavachin isobavachin	2.50	2.38 ± 0.12	4.86	−4.74	2.41 ± 0.08	3.27	−3.51
	250	243.32 ± 5.69	2.34	−2.67	241.75 ± 4.40	1.82	−3.30
	400	382.78 ± 7.18	1.88	−4.30	388.75 ± 6.77	1.74	−2.81
	0.25	0.24 ± 0.01	2.21	−3.73	0.24 ± 0.01	3.34	−4.56
	125	123.55 ± 1.42	1.15	−1.16	123.13 ± 1.29	1.04	−1.50
	200	190.00 ± 4.23	2.23	−5.00	192.54 ± 4.00	2.08	−3.73

Bavachalcone	1.25	1.23 ± 0.02	1.55	−1.76	1.23 ± 0.02	1.31	−1.72
	50	46.32 ± 1.68	3.63	−7.36	46.79 ± 1.39	2.96	−6.41
	80	74.58 ± 2.14	2.86	−6.78	72.93 ± 3.19	4.38	−8.84

Isobavachalcone	2.50	2.39 ± 0.03	1.30	−4.23	2.38 ± 0.07	2.75	−4.96
	250	247.43 ± 1.24	0.50	−1.03	246.79 ± 3.16	1.28	−1.28
	400	392.60 ± 3.72	0.95	−1.85	394.44 ± 3.11	0.79	−1.39

Pinoresinol diglucoside	2.50	2.29 ± 0.12	5.08	−8.39	2.31 ± 0.18	7.89	−7.79
	50	47.09 ± 1.60	3.41	−5.83	46.35 ± 2.48	5.34	−7.29
	80	75.09 ± 2.48	3.31	−6.14	76.13 ± 2.96	3.88	−4.83

Geniposidic acid	2.50	2.42 ± 0.09	3.87	−3.30	2.36 ± 0.17	7.36	−5.56
	1000	958.65 ± 41.85	4.37	−4.14	966.33 ± 43.26	4.48	−3.37
	1600	1571.00 ± 43.79	2.79	−1.81	1,565.67 ± 43.84	2.80	−2.15

Psoralidin	2.50	2.44 ± 0.09	3.79	−2.55	2.41 ± 0.08	3.45	−3.59
	500	487.13 ± 3.63	0.75	−2.57	483.34 ± 6.97	1.44	−3.33
	800	783.10 ± 9.70	1.24	−2.11	787.11 ± 7.81	0.99	−1.61

Neobavaisoflavone	2.50	2.40 ± 0.03	1.23	−3.97	2.42 ± 0.05	2.04	−3.25
	500	471.82 ± 19.17	4.06	−5.64	478.96 ± 14.06	2.94	−4.21
	800	773.13 ± 14.56	1.88	−3.36	772.81 ± 23.72	3.07	−3.40

Bavachinin	2.50	2.27 ± 0.15	6.68	−9.13	2.33 ± 0.15	6.42	−6.68
	250	246.35 ± 5.10	2.07	−1.46	244.05 ± 6.15	2.52	−2.38
	400	387.50 ± 17.19	4.44	−3.13	385.87 ± 12.18	3.16	−3.53

**Table 4:** The extract recoveries and matrix effect of 12 compounds in rat kidney (*n* = 6).

Compound	Concentrations (ng/mL)	Extract recovery (%)	RSD (%)	Matrix effect (%)	RSD (%)
Psoralen	5	87.98 ± 6.41	7.28	94.20 ± 4.04	4.29
	500	87.93 ± 4.88	5.56	93.42 ± 8.77	9.39
	800	85.67 ± 5.13	5.99	90.85 ± 5.01	5.51

Isopsoralen	5	88.74 ± 2.73	3.08	93.86 ± 5.75	6.12
	500	88.60 ± 2.99	3.38	91.92 ± 2.66	2.90
	800	88.99 ± 1.58	1.78	92.27 ± 2.95	3.19

Psoralenoside	12.50	89.79 ± 4.10	4.56	92.47 ± 2.24	2.42
	1000	89.19 ± 0.94	1.06	94.75 ± 0.56	0.59
	1600	85.34 ± 1.60	1.87	94.75 ± 0.71	0.75

Bavachin	2.50	87.75 ± 3.23	3.69	93.05 ± 2.06	2.22
	250	88.84 ± 1.47	1.66	93.96 ± 3.26	3.47
	400	89.48 ± 0.96	1.07	93.99 ± 1.15	1.22

Isobavachin	0.25	88.02 ± 1.93	2.19	93.64 ± 3.13	3.34
	125	85.59 ± 3.12	3.64	95.01 ± 3.74	3.93
	200	88.69 ± 3.70	4.17	96.38 ± 2.98	3.09

Bavachalcone	1.25	89.55 ± 1.41	1.58	94.90 ± 0.60	0.64
	50	88.41 ± 1.30	1.46	91.19 ± 2.13	2.34
	80	89.15 ± 1.08	1.21	91.98 ± 1.05	1.14

Isobavachalcone	2.50	88.16 ± 0.91	1.03	92.54 ± 1.10	1.19
	250	89.37 ± 0.98	1.10	94.42 ± 1.04	1.10
	400	89.85 ± 0.76	0.85	94.79 ± 0.78	0.82

Pinoresinol diglucoside	2.50	84.65 ± 4.01	4.74	94.00 ± 3.43	3.65
	50	88.75 ± 1.09	1.23	94.73 ± 1.00	1.06
	80	88.27 ± 1.42	1.61	93.94 ± 1.45	1.55

Geniposidic acid	2.50	88.32 ± 2.99	3.39	93.39 ± 2.75	2.94
	1000	89.17 ± 0.71	0.79	94.66 ± 1.53	1.62
	1600	90.84 ± 0.97	1.06	98.15 ± 1.89	1.93

Psoralidin	2.50	88.35 ± 2.61	2.95	94.17 ± 1.91	2.03
	500	88.98 ± 1.01	1.14	94.74 ± 1.24	1.31
	800	87.13 ± 3.36	3.85	95.96 ± 0.35	0.36

Psoralen	2.50	88.45 ± 2.73	3.09	93.34 ± 2.25	2.41
	500	81.59 ± 4.60	5.63	88.22 ± 7.30	8.27
	800	85.60 ± 4.27	4.99	94.39 ± 1.09	1.16

Isopsoralen	2.50	89.51 ± 2.68	2.99	91.45 ± 2.94	3.22
	250	87.68 ± 3.15	3.59	94.97 ± 3.76	3.96
	400	85.32 ± 6.02	7.05	93.64 ± 1.86	1.99

**Table 5:** The stability of 12 compounds in rat kidney (*n* = 6).

Compound	Concentrations (ng/mL)	Freeze-thaw stability		Short-term stability		Long-term stability		Preinjection stability	
		Measured (ng/mL)	RE (%)	Measured (ng/mL)	RE (%)	Measured (ng/mL)	RE (%)	Measured (ng/mL)	RE (%)
Psoralen	5	4.90 ± 0.36	−2.00	4.87 ± 0.12	−2.57	4.96 ± 0.21	−0.90	5.08 ± 0.20	1.60
	500	493.95 ± 11.02	−1.21	493.47 ± 13.04	−1.31	501.57 ± 12.64	0.30	497.57 ± 13.3	−0.40
	800	752.75 ± 14.76	−5.91	793.47 ± 13.04	−0.82	802.77 ± 13.39	0.35	787.15 ± 23.6	−1.61

Isopsoralen	5	4.88 ± 0.27	−2.50	4.84 ± 0.16	−3.23	4.93 ± 0.21	−1.40	4.98 ± 0.12	−0.37
	500	493.12 ± 9.04	−1.38	493.47 ± 13.04	−1.31	498.57 ± 10.47	−0.29	495.07 ± 11.99	−0.99
	800	759.58 ± 18.59	−5.05	790.80 ± 13.58	−1.15	801.77 ± 8.19	0.22	792.32 ± 12.03	−0.96

Psoralenoside	12.50	12.30 ± 0.29	−1.60	12.40 ± 0.10	−0.81	12.27 ± 0.10	−1.85	12.37 ± 0.12	−1.03
	1000	988.95 ± 11.05	−1.11	990.13 ± 6.60	−0.99	973.80 ± 16.36	−2.62	977.79 ± 12.29	−2.22
	1600	1577.75 ± 16.66	−1.39	1586.80 ± 7.10	−0.83	1578.47 ± 7.69	−1.35	1581.0 ± 8.50	−1.19

Bavachin	2.50	2.41 ± 0.06	−3.67	2.36 ± 0.09	−5.45	2.40 ± 0.05	−4.00	2.45 ± 0.05	−2.00
	250	243.18 ± 4.68	−2.73	242.37 ± 8.80	−3.05	239.33 ± 7.01	−4.27	247.38 ± 3.65	−1.05
	400	384.08 ± 12.05	−3.98	386.93 ± 10.07	−3.27	386.17 ± 5.17	−3.46	391.00 ± 5.33	−2.25

Isobavachin	0.25	0.24 ± 0.01	−3.67	0.24 ± 0.01	−5.37	0.24 ± 0.00	−4.4	0.24 ± 0.01	−4.42
	125	123.35 ± 1.13	−1.32	124.37 ± 0.52	−0.51	123.50 ± 0.98	−1.20	123.38 ± 1.89	−1.29
	200	197.08 ± 2.84	−1.46	195.08 ± 3.07	−2.46	193.33 ± 2.61	−3.33	196.00 ± 4.29	−2.00

Bavachalcone	1.25	1.24 ± 0.01	−0.73	1.23 ± 0.02	−1.87	1.24 ± 0.01	−1.17	1.23 ± 0.01	−1.27
	50	48.40 ± 1.00	−3.2	46.37 ± 1.93	−7.27	45.33 ± 1.97	−9.33	47.32 ± 1.82	−5.37
	80	77.08 ± 2.84	−3.65	76.42 ± 2.77	−4.84	75.00 ± 2.63	−6.25	78.50 ± 3.02	−1.88

Isobavachalcone	2.50	2.42 ± 0.03	−3.01	2.45 ± 0.05	−2.09	2.43 ± 0.05	−2.93	2.46 ± 0.05	−1.80
	250	242.18 ± 2.93	−3.13	244.85 ± 5.63	−2.06	242.83 ± 3.72	−2.87	247.78 ± 3.67	−0.89
	400	388.97 ± 7.41	−2.76	391.27 ± 6.48	−2.18	390.72 ± 6.54	−2.32	391.50 ± 5.85	−2.13

Pinoresinol diglucoside	2.50	2.40 ± 0.07	−4.13	2.43 ± 0.03	−2.67	2.46 ± 0.09	−1.73	2.39 ± 0.12	−4.53
	50	49.28 ± 0.59	−1.43	49.30 ± 0.77	−1.40	48.90 ± 1.09	−2.20	49.57 ± 1.35	−0.87
	80	77.25 ± 1.68	−3.44	79.23 ± 0.64	−0.97	79.34 ± 0.85	−0.82	78.98 ± 1.60	−1.27
	2.5	2.31 ± 0.19	−7.41	2.26 ± 0.11	−0.95	2.28 ± 0.16	−0.87	2.26 ± 0.18	−0.64
	1000	969.67 ± 32.73	−3.03	942.67 ± 22.73	−5.73	980.50 ± 24.99	−1.95	923.63 ± 25.24	−7.64
	1600	1562.83 ± 39.05	−2.32	1575.83 ± 39.84	−1.51	1570.33 ± 37.45	−1.85	1583.33 ± 24.27	−1.04

Psoralidin	2.50	2.42 ± 0.04	−3.27	2.46 ± 0.04	−1.53	2.45 ± 0.05	−2.20	2.45 ± 0.05	−2.20
	500	493.12 ± 8.33	−1.37	494.30 ± 7.37	−1.14	497.23 ± 14.14	−0.55	498.57 ± 12.55	−0.29
	800	777.75 ± 9.36	−2.78	797.47 ± 5.18	−0.32	801.10 ± 8.14	0.14	791.62 ± 16.58	−1.05

Psoralen	2.50	2.42 ± 0.06	−3.02	2.27 ± 0.13	−9.19	2.36 ± 0.10	−5.73	2.38 ± 0.11	−4.63
	500	467.93 ± 17.07	−6.41	470.27 ± 25.24	−5.95	485.50 ± 19.43	−2.90	466.13 ± 28.44	−6.77
	800	760.12 ± 25.96	−4.99	782.12 ± 22.41	−2.24	776.95 ± 17.25	−2.88	768.48 ± 19.69	−3.94

Isopsoralen	2.50	2.40 ± 0.09	−3.87	2.27 ± 0.16	−9.02	2.39 ± 0.13	−4.31	2.42 ± 0.09	−3.26
	250	243.68 ± 5.47	−2.53	246.87 ± 18.29	−1.25	235.50 ± 10.69	−5.80	246.55 ± 8.19	−1.38
	400	379.92 ± 27.37	−5.02	382.93 ± 24.86	−4.27	384.83 ± 12.14	−3.79	389.33 ± 12.53	−2.67

## Data Availability

The main data used to support the findings of this study are included within the article. The methodological data for organs other than kidney's data used to support the findings of this study are included within the supplementary information file.

## Abbreviations

TEM: Ion source temperature
CUR: Curtain gas
GAS: Ion source gas
MRM: Multiple reactions monitoring
DP: Declustering potential
CE: Collision energy
AUC: Area under the curve
ESI: Information-dependent acquisition
IS: Internal standard
LLOD: The lower limit of detection
LLOQ: The lower limit of quantitation
QC: Quality control
RTE: Relative targeting efficiency
SD: Sprague-Dawley
TCM: Traditional Chinese medicine
UHPLC-MS/MS: Ultrahigh-performance liquid chromatography-tandem mass spectrometry.

## Supplementary Materials

Supplementary Materials The supplementary material [23] we submitted is methodological data of 12 compounds in the heart, liver, spleen, lung, uterus, testes, and ovary. The results of precision and accuracy for the heart, liver, spleen, lung, uterus, ovary, and testes are shown in Tables 1–7. The extract recoveries and matrix effect in the heart, liver, spleen, lung, uterus, ovary, and testes are shown in Tables 8–14. The results of compound stability in the heart, liver, spleen, lung, uterus, ovary, and testes tissue under different storage conditions are presented in Tables 15–21.
