# Supplementary material for: Establishment of a UPLC-MS/MS Method for Studying the Effect of Salt-Processing on Tissue Distribution of Twelve Major Bioactive Components of Qing'e Pills in Rats
Source: J Anal Methods Chem. 2020 Sep 17;2020:8832736. doi: 10.1155/2020/8832736 (PMC7519442; doi:10.1155/2020/8832736)
Supplement: Supplementary Materials — The supplementary material [23] we submitted is methodological data of 12 compounds in the heart, liver, spleen, lung, uterus, testes, and ovary. The results of precision and accuracy for the heart, liver, spleen, lung, uterus, ovary, and testes are shown in Tables 1–7. The extract recoveries and matrix effect in the heart, liver, spleen, lung, uterus, ovary, and testes are shown in Tables 8–14. The results of compound stability in the heart, liver, spleen, lung, uterus, ovary, and testes tissue under different storage conditions are presented in Tables 15–21. [file 8832736.f1.doc]

**Methodology attachment**

**Precision and Accuracy**

The results for the heart, liver, spleen, lung, uterus, ovary, and testes are shown in Table 1–7. The accuracy and precision of the method were within the specified range, meeting the requirements for biological sample determination.

**Table 1.** Intra-assay and inter-assay precision, accuracy of 12 compounds in rat heart (*n* = 6).

| Compound | Concentrations  (ng/mL) |  | Intraday |  |  | Interday |  |
| --- | --- | --- | --- | --- | --- | --- | --- |
|  |  | Measured  (ng/mL) | Precision(%) | Accuracy(%) | Measured  (ng/mL) | Precision (%) | Accuracy(%) |
| Psoralen | 2.5 | 2.45±0.04 | 1.54 | -2.07 | 2.41±0.09 | 3.61 | -3.58 |
|  | 500 | 474.15±32.69 | 6.89 | -5.17 | 481.42±23.16 | 4.81 | -3.72 |
|  | 800 | 747.65±7.47 | 1.00 | -6.54 | 758.87±17.27 | 2.28 | -5.14 |
| Isopsoralen | 2.5 | 2.38±0.07 | 2.87 | -4.92 | 2.39±0.19 | 8.10 | -4.29 |
|  | 500 | 472.25±2.74 | 0.58 | -5.55 | 455.94±20.90 | 4.58 | -8.81 |
|  | 800 | 727.63±37.24 | 5.12 | -9.05 | 713.44±23.11 | 3.24 | -10.82 |
| Psoralenoside | 12.5 | 12.00±0.43 | 3.60 | -4.03 | 11.74±0.45 | 3.87 | -6.09 |
|  | 500 | 461.72±1.67 | 0.36 | -7.66 | 459.05±14.70 | 3.20 | -8.19 |
|  | 800 | 704.35±5.91 | 0.84 | -11.96 | 732.23±30.73 | 4.20 | -8.47 |
| Bavachin | 1 | 0.97±0.02 | 2.20 | -2.78 | 0.95±0.03 | 2.91 | -5.03 |
|  | 25 | 23.47±1.53 | 6.50 | -6.11 | 23.08±1.31 | 5.67 | -7.68 |
|  | 40 | 36.73±3.05 | 8.30 | -8.17 | 36.80±2.70 | 7.34 | -7.99 |
| Isobavachin | 1 | 0.96±0.03 | 2.74 | -3.67 | 0.93±0.05 | 5.19 | -6.92 |
|  | 25 | 23.97±0.77 | 3.21 | -4.13 | 22.98±1.68 | 7.33 | -8.09 |
|  | 40 | 38.26±0.79 | 2.07 | -4.36 | 35.26±2.66 | 7.54 | -11.85 |
| Bavachalcone | 1 | 0.96±0.02 | 1.93 | -3.94 | 0.95±0.03 | 3.09 | -4.70 |
|  | 12.5 | 11.64±0.41 | 3.49 | -6.89 | 11.63±0.38 | 3.31 | -6.99 |
|  | 20 | 18.14±0.82 | 4.52 | -9.31 | 17.55±1.37 | 7.79 | -12.24 |
| Isobavachalcone | 2.5 | 2.43±0.06 | 2.32 | -2.73 | 2.39±0.07 | 3.06 | -4.33 |
|  | 25 | 22.89±0.94 | 4.12 | -8.44 | 23.33±0.79 | 3.39 | -6.66 |
|  | 40 | 35.77±3.32 | 9.29 | -10.57 | 37.01±2.08 | 5.61 | -7.48 |
| Pinoresinol diglucoside | 1.25 | 1.15±0.04 | 3.34 | -8.16 | 1.14±0.05 | 4.59 | -8.63 |
|  | 50 | 45.27±2.73 | 6.04 | -9.45 | 45.56±3.14 | 6.90 | -8.89 |
|  | 80 | 71.45±4.79 | 6.70 | -10.69 | 72.38±5.33 | 7.36 | -9.53 |
| Geniposidic acid | 2.5 | 2.45±0.04 | 1.61 | -2.02 | 2.37±0.08 | 3.57 | -5.20 |
|  | 250 | 241.77±3.98 | 1.65 | -3.29 | 235.72±9.33 | 3.96 | -5.71 |
|  | 400 | 376.78±13.06 | 3.47 | -5.80 | 375.88±15.94 | 4.24 | -6.03 |
| Psoralidin | 2.5 | 2.46±0.04 | 1.58 | -1.71 | 2.35±0.11 | 4.86 | -5.82 |
|  | 125 | 117.94±3.65 | 3.09 | -5.65 | 116.28±6.17 | 5.31 | -6.98 |
|  | 200 | 181.78±5.63 | 3.10 | -9.11 | 184.10±10.75 | 5.84 | -7.95 |
| Neobavaisoflavone | 1.25 | 1.21±0.02 | 1.76 | -3.52 | 1.19±0.03 | 2.25 | -4.98 |
|  | 25 | 22.94±1.35 | 5.87 | -8.24 | 22.33±1.50 | 6.73 | -10.66 |
|  | 40 | 34.95±2.48 | 7.10 | -12.62 | 34.54±3.09 | 8.93 | -13.64 |
| Bavachinin | 2.5 | 2.38±0.08 | 3.21 | -4.80 | 2.38±0.07 | 2.99 | -4.85 |
|  | 50 | 48.11±1.19 | 2.47 | -3.79 | 46.95±2.08 | 4.43 | -6.11 |
|  | 80 | 74.78±2.80 | 3.75 | -6.52 | 75.10±2.68 | 3.57 | -6.13 |

**Table 2.** Intra-assay and inter-assay precision, accuracy of 12 compounds in rat liver (*n* = 6).

| Compound | Concentrations(ng/mL) |  | Intraday |  |  | Interday |  |
| --- | --- | --- | --- | --- | --- | --- | --- |
|  |  | Measured  (ng/mL) | Precision(%) | Accuracy(%) | Measured  (ng/mL) | Precision (%) | Accuracy(%) |
| Psoralen | 12.5 | 12.26±0.08 | 0.69 | -1.90 | 12.24±0.08 | 0.67 | -2.06 |
|  | 500 | 481.29±6.19 | 1.29 | -3.74 | 485.56±9.73 | 2.00 | -2.89 |
|  | 800 | 763.45±10.15 | 1.33 | -4.57 | 763.54±19.53 | 2.56 | -4.56 |
| Isopsoralen | 12.5 | 12.21±0.02 | 0.14 | -2.29 | 12.23±0.06 | 0.49 | -2.15 |
|  | 500 | 485.27±12.65 | 2.61 | -2.95 | 484.83±11.31 | 2.33 | -3.03 |
|  | 800 | 770.12±28.48 | 3.70 | -3.74 | 767.43±25.30 | 3.30 | -4.07 |
| Psoralenoside | 12.5 | 12.41±0.07 | 0.57 | -0.71 | 12.31±0.11 | 0.90 | -1.55 |
|  | 500 | 486.22±8.59 | 1.77 | -2.76 | 467.21±20.60 | 4.41 | -6.56 |
|  | 800 | 736.02±27.80 | 3.78 | -8.00 | 742.78±27.83 | 3.75 | -7.15 |
| Bavachin | 2.5 | 2.41±0.05 | 2.11 | -3.56 | 2.41±0.04 | 1.84 | -3.69 |
|  | 250 | 237.22±8.19 | 3.45 | -5.11 | 238.66±7.69 | 3.22 | -4.54 |
|  | 400 | 376.78±13.06 | 3.47 | -5.80 | 363.18±20.78 | 5.72 | -9.20 |
| Isobavachin | 2.5 | 2.44±0.04 | 1.59 | -2.31 | 2.38±0.08 | 3.38 | -4.92 |
|  | 125 | 119.61±2.78 | 2.32 | -4.31 | 117.83±5.41 | 4.59 | -5.73 |
|  | 200 | 186.78±5.29 | 2.83 | -6.61 | 181.88±9.25 | 5.08 | -9.06 |
| Bavachalcone | 1.25 | 1.22±0.02 | 1.38 | -2.79 | 1.22±0.02 | 1.36 | -2.31 |
|  | 125 | 117.44±5.07 | 4.32 | -6.05 | 117.50±4.29 | 3.65 | -6.00 |
|  | 200 | 182.95±14.52 | 7.94 | -8.52 | 181.16±10.39 | 5.74 | -9.42 |
| Isobavachalcone | 2.5 | 2.41±0.08 | 3.18 | -3.71 | 2.38±0.08 | 3.41 | -4.87 |
|  | 50 | 45.44±2.22 | 4.89 | -9.12 | 45.17±2.60 | 5.76 | -9.66 |
|  | 80 | 68.95±5.24 | 7.60 | -13.81 | 71.10±4.54 | 6.38 | -11.13 |
| Pinoresinol diglucoside | 1.25 | 1.22±0.02 | 1.48 | -2.37 | 1.22±0.01 | 1.17 | -2.21 |
|  | 125 | 118.94±2.95 | 2.48 | -4.85 | 117.28±3.90 | 3.33 | -6.18 |
|  | 200 | 181.78±6.90 | 3.80 | -9.11 | 179.10±18.54 | 10.35 | -10.45 |
| Geniposidic acid | 2.5 | 2.43±0.04 | 1.75 | -2.82 | 2.45±0.04 | 1.66 | -2.10 |
|  | 500 | 476.44±12.58 | 2.64 | -4.71 | 474.28±17.96 | 3.79 | -5.14 |
|  | 800 | 749.28±35.48 | 4.74 | -6.34 | 749.93±29.36 | 3.92 | -6.26 |
| Psoralidin | 2.5 | 2.45±0.06 | 2.56 | -2.15 | 2.40±0.08 | 3.25 | -4.00 |
|  | 250 | 239.77±7.36 | 3.07 | -4.09 | 234.39±11.30 | 4.82 | -6.24 |
|  | 400 | 376.78±13.06 | 3.47 | -5.80 | 374.10±21.44 | 5.73 | -6.48 |
| Neobavaisoflavone | 2.5 | 2.45±0.04 | 1.56 | -2.07 | 2.45±0.03 | 1.37 | -1.90 |
|  | 500 | 235.44±5.55 | 2.36 | -5.82 | 237.45±7.39 | 3.11 | -5.02 |
|  | 800 | 371.78±12.69 | 3.41 | -7.05 | 374.10±15.83 | 4.23 | -6.48 |
| Bavachinin | 2.5 | 2.47±0.02 | 0.99 | -1.10 | 2.45±0.04 | 1.54 | -1.86 |
|  | 500 | 480.94±8.78 | 1.83 | -3.81 | 478.67±16.07 | 3.36 | -4.27 |
|  | 800 | 768.45±25.00 | 3.25 | -3.94 | 759.10±29.73 | 3.92 | -5.11 |

**Table 3.** Intra-assay and inter-assay precision, accuracy of 12 compounds in rat spleen (*n* = 6).

| Compound | Concentrations(ng/mL) |  | Intraday |  |  | Interday |  |
| --- | --- | --- | --- | --- | --- | --- | --- |
|  |  | Measured  (ng/mL) | Precision(%) | Accuracy(%) | Measured  (ng/mL) | Precision (%) | Accuracy(%) |
| Psoralen | 12.5 | 12.45±0.03 | 0.26 | -0.40 | 12.44±0.03 | 0.26 | -0.46 |
|  | 500 | 488.69±9.29 | 1.90 | -2.26 | 485.16±9.63 | 1.98 | -2.97 |
|  | 800 | 761.46±27.98 | 3.67 | -4.82 | 760.85±27.72 | 3.64 | -4.89 |
| Isopsoralen | 12.5 | 12.45±0.03 | 0.23 | -0.38 | 12.44±0.03 | 0.25 | -0.47 |
|  | 500 | 488.52±8.85 | 1.81 | -2.30 | 483.99±10.35 | 2.14 | -3.20 |
|  | 800 | 761.46±29.35 | 3.85 | -4.82 | 757.51±30.69 | 4.05 | -5.31 |
| Psoralenoside | 12.5 | 12.43±0.04 | 0.33 | -0.53 | 12.43±0.04 | 0.29 | -0.54 |
|  | 500 | 485.19±12.79 | 2.64 | -2.96 | 483.49±10.91 | 2.26 | -3.30 |
|  | 800 | 761.46±31.22 | 4.10 | -4.82 | 759.18±30.21 | 3.98 | -5.10 |
| Bavachin | 2.5 | 2.43±0.05 | 2.07 | -2.70 | 2.44±0.04 | 1.61 | -2.37 |
|  | 100 | 93.02±3.71 | 3.99 | -6.98 | 91.60±4.87 | 5.31 | -8.40 |
|  | 160 | 146.12±7.80 | 5.34 | -8.67 | 146.29±11.66 | 7.97 | -8.57 |
| Isobavachin | 1.25 | 1.23±0.03 | 2.22 | -1.74 | 1.23±0.02 | 1.66 | -1.39 |
|  | 50 | 46.52±3.93 | 8.45 | -6.96 | 46.10±3.23 | 7.02 | -7.80 |
|  | 80 | 73.12±4.82 | 6.60 | -8.60 | 73.46±3.93 | 5.35 | -8.18 |
| Bavachalcone | 1.25 | 1.22±0.02 | 1.24 | -2.33 | 1.22±0.01 | 1.04 | -2.36 |
|  | 25 | 23.35±0.85 | 3.65 | -6.58 | 22.99±1.18 | 5.15 | -8.04 |
|  | 40 | 36.12±3.23 | 8.93 | -9.69 | 36.01±3.24 | 8.99 | -9.96 |
| Isobavachalcone | 1.25 | 1.24±0.00 | 0.08 | -0.52 | 1.25±0.04 | 3.06 | -0.32 |
|  | 125 | 121.02±2.93 | 2.42 | -3.18 | 120.94±2.64 | 2.18 | -3.25 |
|  | 200 | 180.62±7.66 | 4.24 | -9.69 | 181.29±5.96 | 3.29 | -9.35 |
| Pinoresinol diglucoside | 1.25 | 1.22±0.02 | 1.53 | -2.33 | 1.22±0.01 | 1.14 | -2.15 |
|  | 50 | 47.02±2.76 | 5.87 | -5.96 | 45.60±2.91 | 6.38 | -8.80 |
|  | 80 | 76.46±4.42 | 5.78 | -4.43 | 75.29±3.58 | 4.75 | -5.88 |
| Geniposidic acid | 2.5 | 2.44±0.02 | 0.99 | -2.31 | 2.45±0.03 | 1.05 | -2.17 |
|  | 250 | 243.02±5.56 | 2.29 | -2.79 | 241.05±5.82 | 2.41 | -3.58 |
|  | 400 | 383.12±7.64 | 2.00 | -4.22 | 380.29±8.21 | 2.16 | -4.93 |
| Psoralidin | 2.5 | 2.42±0.01 | 0.49 | -3.18 | 2.44±0.03 | 1.30 | -2.41 |
|  | 250 | 243.69±5.37 | 2.20 | -2.52 | 243.10±3.86 | 1.59 | -2.76 |
|  | 400 | 386.96±10.38 | 2.68 | -3.26 | 387.13±6.88 | 1.78 | -3.22 |
| Neobavaisoflavone | 2.5 | 2.44±0.03 | 1.24 | -2.33 | 2.45±0.03 | 1.15 | -2.12 |
|  | 250 | 243.85±5.40 | 2.21 | -2.46 | 237.99±8.55 | 3.59 | -4.80 |
|  | 400 | 383.12±7.98 | 2.08 | -4.22 | 380.85±8.85 | 2.32 | -4.79 |
| Bavachinin | 2.5 | 2.45±0.04 | 1.47 | -1.84 | 2.44±0.03 | 1.39 | -2.20 |
|  | 500 | 490.35±6.32 | 1.29 | -1.93 | 490.16±5.67 | 1.16 | -1.97 |
|  | 800 | 784.79±8.11 | 1.03 | -1.90 | 779.74±8.82 | 1.13 | -2.53 |

**Table 4.** Intra-assay and inter-assay precision, accuracy of 12 compounds in rat lung (*n* = 6).

| Compound | Concentrations(ng/mL) |  | Intraday |  |  | Interday |  |
| --- | --- | --- | --- | --- | --- | --- | --- |
|  |  | Measured  (ng/mL) | Precision(%) | Accuracy(%) | Measured  (ng/mL) | Precision (%) | Accuracy(%) |
| Psoralen | 12.5 | 12.22±0.59 | 4.83 | -2.28 | 12.57±0.85 | 6.79 | 0.55 |
|  | 500 | 486.92±22.43 | 4.61 | -2.62 | 482.78±25.77 | 5.34 | -3.44 |
|  | 800 | 720.27±33.64 | 4.67 | -9.97 | 741.78±31.49 | 4.24 | -7.28 |
| Isopsoralen | 12.5 | 12.09±0.64 | 5.31 | -3.31 | 12.28±0.74 | 6.01 | -1.80 |
|  | 500 | 481.45±10.48 | 2.18 | -3.71 | 496.59±16.42 | 3.31 | -0.68 |
|  | 800 | 752.97±23.53 | 3.13 | -5.88 | 757.17±28.90 | 3.82 | -5.35 |
| Psoralenoside | 2.5 | 2.45±0.05 | 2.23 | -2.06 | 2.44±0.07 | 2.67 | -2.30 |
|  | 1000 | 970.08±19.27 | 1.99 | -2.99 | 977.42±23.86 | 2.44 | -2.26 |
|  | 1600 | 1420.50±77.80 | 5.48 | -11.22 | 1469.17±94.10 | 6.40 | -8.18 |
| Bavachin | 1.25 | 1.25±0.01 | 0.45 | -0.35 | 1.24±0.01 | 0.45 | -0.53 |
|  | 100 | 98.10±3.98 | 4.06 | -1.90 | 98.23±7.00 | 7.13 | -1.77 |
|  | 160 | 153.65±5.85 | 3.80 | -3.97 | 150.98±5.35 | 3.54 | -5.64 |
| Isobavachin | 1.25 | 1.24±0.01 | 0.43 | -0.93 | 1.24±0.01 | 0.59 | -1.00 |
|  | 50 | 48.79±3.99 | 8.19 | -2.43 | 47.64±3.33 | 7.00 | -4.71 |
|  | 80 | 76.10±6.72 | 8.83 | -4.87 | 77.66±5.12 | 6.59 | -2.92 |
| Bavachalcone | 1.25 | 1.24±0.01 | 1.04 | -0.52 | 1.25±0.01 | 0.65 | -0.26 |
|  | 50 | 47.98±1.32 | 2.75 | -4.03 | 50.52±2.32 | 4.59 | 1.03 |
|  | 80 | 80.12±0.61 | 0.76 | 0.00 | 81.18±2.10 | 2.59 | 1.47 |
| Isobavachalcone | 2.5 | 1.25±0.00 | 0.22 | -0.32 | 1.25±0.00 | 0.37 | -0.35 |
|  | 12.5 | 11.46±0.24 | 2.09 | -8.36 | 11.57±0.34 | 2.90 | -7.42 |
|  | 200 | 193.20±11.05 | 5.72 | -3.40 | 198.69±7.73 | 3.89 | -0.66 |
| Pinoresinol diglucoside | 1.25 | 1.25±0.01 | 0.44 | -0.13 | 1.24±0.01 | 0.57 | -0.40 |
|  | 250 | 247.65±7.71 | 3.11 | -0.94 | 243.01±7.90 | 3.25 | -2.80 |
|  | 400 | 376.12±31.67 | 8.42 | -5.97 | 391.66±37.34 | 9.53 | -2.08 |
| Geniposidic acid | 2.5 | 2.49±0.02 | 0.74 | -0.59 | 2.48±0.02 | 0.84 | -0.60 |
|  | 500 | 496.25±11.34 | 2.29 | -0.75 | 491.03±20.44 | 4.16 | -1.79 |
|  | 800 | 787.83±32.86 | 4.17 | -1.52 | 784.72±36.02 | 4.59 | -1.91 |
| Psoralidin | 2.5 | 2.45±0.08 | 3.33 | -1.94 | 2.44±0.10 | 4.00 | -2.37 |
|  | 250 | 259.06±15.25 | 5.89 | 3.62 | 247.56±16.07 | 6.49 | -0.98 |
|  | 400 | 387.33±5.10 | 1.32 | -3.17 | 386.44±9.12 | 2.36 | -3.39 |
| Neobavaisoflavone | 2.5 | 2.47±0.03 | 1.30 | -1.11 | 2.42±0.08 | 3.46 | -0.79 |
|  | 150 | 147.00±3.54 | 2.41 | -2.00 | 150.24±4.53 | 3.01 | 0.16 |
|  | 240 | 232.26±5.92 | 2.55 | -3.22 | 228.90±13.34 | 5.83 | -4.63 |
| Bavachinin | 2.5 | 2.45±0.18 | 7.16 | -2.05 | 2.41±0.16 | 6.60 | -3.48 |
|  | 250 | 248.58±11.85 | 4.77 | -0.57 | 245.00±11.19 | 4.57 | -2.00 |
|  | 400 | 395.27±9.89 | 2.50 | -1.18 | 404.34±28.71 | 7.10 | 1.08 |

**Table 5.** Intra-assay and inter-assay precision, accuracy of 12 compounds in rat uterus (*n* = 6).

| Compound | Concentrations(ng/mL) |  | Intraday |  |  | Interday |  |
| --- | --- | --- | --- | --- | --- | --- | --- |
|  |  | Measured  (ng/mL) | Precision(%) | Accuracy(%) | Measured  (ng/mL) | Precision (%) | Accuracy  (%) |
| Psoralen | 2.5 | 2.34±0.13 | 5.34 | -6.23 | 2.37±0.20 | 8.26 | -5.27 |
|  | 500 | 502.15±15.75 | 3.14 | 0.43 | 487.67±16.45 | 3.37 | -2.47 |
|  | 800 | 778.20±23.23 | 2.99 | -2.73 | 787.58±22.05 | 2.80 | -1.55 |
| Isopsoralen | 2.5 | 2.34±0.09 | 3.91 | -6.53 | 2.41±0.10 | 4.07 | -3.57 |
|  | 250 | 248.27±13.31 | 5.36 | -0.69 | 247.65±10.13 | 4.09 | -0.94 |
|  | 400 | 384.03±24.06 | 6.27 | -3.99 | 390.44±17.50 | 4.48 | -2.39 |
| Psoralenoside | 12.5 | 12.44±0.05 | 0.40 | -0.45 | 12.44±0.06 | 0.52 | -0.46 |
|  | 1000 | 989.58±13.66 | 1.38 | -1.04 | 991.15±13.15 | 1.33 | -0.88 |
|  | 1600 | 1575.61±29.83 | 1.89 | -1.52 | 1582.99±22.12 | 1.40 | -1.06 |
| Bavachin | 2.5 | 2.48±0.09 | 3.64 | -0.65 | 2.47±0.08 | 3.30 | -1.33 |
|  | 100 | 98.42±4.74 | 4.82 | -1.58 | 99.19±3.78 | 3.81 | -0.81 |
|  | 160 | 156.95±8.47 | 5.40 | -1.91 | 155.92±9.51 | 6.10 | -2.55 |
| Isobavachin | 0.25 | 0.24±0.01 | 2.12 | -2.67 | 0.24±0.01 | 2.48 | -3.00 |
|  | 25 | 24.42±1.17 | 4.78 | -2.33 | 24.55±1.02 | 4.15 | -1.80 |
|  | 40 | 39.93±2.47 | 6.20 | -0.17 | 39.88±2.15 | 5.38 | -0.30 |
| Bavachalcone | 1.25 | 1.24±0.01 | 0.51 | -0.61 | 1.24±0.01 | 0.71 | -0.52 |
|  | 125 | 124.21±1.37 | 1.11 | -0.63 | 124.60±2.08 | 1.67 | -0.32 |
|  | 200 | 197.91±10.68 | 5.40 | -1.05 | 197.80±10.27 | 5.19 | -1.10 |
| Isobavachalcone | 2.5 | 2.48±0.04 | 1.64 | -0.87 | 2.48±0.04 | 1.75 | -0.96 |
|  | 25 | 24.94±1.38 | 5.52 | -0.25 | 24.79±1.09 | 4.41 | -0.84 |
|  | 40 | 39.82±3.34 | 8.38 | -0.45 | 39.08±3.57 | 9.12 | -2.29 |
| Pinoresinol diglucoside | 1.25 | 1.24±0.01 | 0.94 | -0.67 | 1.24±0.01 | 1.04 | -0.49 |
|  | 50 | 49.84±0.90 | 1.80 | -0.32 | 49.82±1.94 | 3.90 | -0.36 |
|  | 80 | 79.91±4.00 | 5.00 | -0.11 | 79.59±4.11 | 5.17 | -0.52 |
| Geniposidic acid | 1.25 | 1.24±0.01 | 0.83 | -0.53 | 1.24±0.01 | 0.82 | -0.58 |
|  | 250 | 249.59±3.59 | 1.44 | -0.16 | 249.37±4.28 | 1.72 | -0.25 |
|  | 400 | 393.68±14.10 | 3.58 | -1.58 | 396.38±18.28 | 4.61 | -0.91 |
| Psoralidin | 2.5 | 2.49±0.03 | 1.04 | -0.53 | 2.47±0.03 | 1.24 | -1.02 |
|  | 125 | 124.75±2.44 | 1.96 | -0.20 | 123.58±3.60 | 2.91 | -1.14 |
|  | 200 | 197.54±9.45 | 4.78 | -1.23 | 195.73±10.36 | 5.29 | -2.14 |
| Neobavaisoflavone | 2.5 | 2.48±0.05 | 2.18 | -0.73 | 2.48±0.04 | 1.51 | -0.89 |
|  | 25 | 24.32±0.73 | 2.99 | -2.73 | 24.39±0.66 | 2.69 | -2.45 |
|  | 40 | 37.29±1.88 | 5.04 | -6.78 | 38.26±1.76 | 4.60 | -4.36 |
| Bavachinin | 2.5 | 2.49±0.02 | 0.79 | -0.53 | 2.48±0.02 | 0.76 | -0.62 |
|  | 25 | 24.54±0.41 | 1.66 | -1.84 | 24.65±0.39 | 1.58 | -1.40 |
|  | 40 | 38.96±1.43 | 3.66 | -2.59 | 39.00±1.40 | 3.59 | -2.51 |

**Table 6.** Intra-assay and inter-assay precision, accuracy of 12 compounds in rat t ovary (*n* = 6).

| Compound | Concentrations(ng/mL) |  | Intraday |  |  | Interday |  |
| --- | --- | --- | --- | --- | --- | --- | --- |
|  |  | Measured  (ng/mL) | Precision(%) | Accuracy(%) | Measured  (ng/mL) | Precision (%) | Accuracy(%) |
| Psoralen | 12.5 | 12.48±0.12 | 0.95 | -0.13 | 12.45±0.17 | 1.33 | -0.40 |
|  | 500 | 493.90±19.22 | 3.89 | -1.22 | 495.07±15.97 | 3.23 | -0.99 |
|  | 800 | 785.92±26.12 | 3.32 | -1.76 | 788.26±25.32 | 3.21 | -1.47 |
| Isopsoralen | 12.5 | 12.32±0.39 | 3.16 | -1.48 | 12.42±0.30 | 2.38 | -0.64 |
|  | 500 | 494.78±21.80 | 4.41 | -1.04 | 495.15±18.70 | 3.78 | -0.97 |
|  | 800 | 787.87±35.03 | 4.45 | -1.52 | 790.16±28.83 | 3.65 | -1.23 |
| Psoralenoside | 12.5 | 12.43±0.50 | 3.99 | -0.55 | 12.37±0.45 | 3.61 | -1.05 |
|  | 1000 | 990.00±68.77 | 6.95 | -1.00 | 983.09±68.79 | 7.00 | -1.69 |
|  | 1600 | 1590.33±92.26 | 5.80 | -0.60 | 1589.22±86.45 | 5.44 | -0.67 |
| Bavachin | 2.5 | 2.48±0.09 | 3.45 | -0.80 | 2.46±0.07 | 2.72 | -75.39 |
|  | 25 | 24.27±1.17 | 4.82 | -2.91 | 24.40±1.06 | 4.34 | -75.60 |
|  | 40 | 39.45±2.09 | 5.29 | -1.38 | 39.45±2.17 | 5.51 | -90.14 |
| Isobavachin | 2.5 | 2.48±0.10 | 4.05 | -0.87 | 2.48±0.07 | 2.98 | -0.69 |
|  | 25 | 24.38±1.11 | 4.56 | -2.47 | 24.55±1.20 | 4.88 | -1.80 |
|  | 40 | 39.43±2.13 | 5.41 | -1.42 | 38.87±2.08 | 5.36 | -2.82 |
| Bavachalcone | 1.25 | 1.25±0.01 | 0.97 | 0.27 | 1.24±0.02 | 1.76 | -0.76 |
|  | 25 | 24.40±1.53 | 6.25 | -2.40 | 24.45±1.27 | 5.19 | -2.20 |
|  | 40 | 38.03±2.71 | 7.13 | -4.92 | 38.66±2.58 | 6.68 | -3.35 |
| Isobavachalcone | 1.25 | 1.24±0.05 | 4.21 | -0.80 | 1.24±0.03 | 2.79 | -1.07 |
|  | 25 | 24.45±1.25 | 5.10 | -2.20 | 24.22±1.28 | 5.27 | -3.13 |
|  | 40 | 38.13±2.01 | 5.28 | -4.67 | 38.91±2.33 | 5.98 | -2.73 |
| Pinoresinol diglucoside | 1.25 | 1.24±0.01 | 1.14 | -0.80 | 1.24±0.03 | 2.03 | -0.78 |
|  | 50 | 49.53±1.66 | 3.36 | -0.93 | 48.93±2.49 | 5.10 | -2.13 |
|  | 80 | 78.73±3.03 | 3.84 | -1.58 | 78.77±3.32 | 4.21 | -1.54 |
| Geniposidic acid | 2.5 | 2.49±0.02 | 0.83 | -0.60 | 2.48±0.03 | 1.33 | -0.69 |
|  | 500 | 497.58±18.14 | 3.65 | -0.48 | 491.05±18.16 | 3.70 | -1.79 |
|  | 800 | 790.77±25.68 | 3.25 | -1.15 | 790.53±24.53 | 3.10 | -1.18 |
| Psoralidin | 1.25 | 1.24±0.04 | 3.26 | -0.47 | 1.24±0.02 | 1.99 | -0.64 |
|  | 50 | 49.31±1.38 | 2.79 | -1.37 | 49.40±1.18 | 2.39 | -1.20 |
|  | 80 | 79.38±1.99 | 2.51 | -0.77 | 79.53±1.93 | 2.42 | -0.58 |
| Neobavaisoflavone | 2.5 | 2.48±0.02 | 0.83 | -0.67 | 2.48±0.02 | 0.68 | -0.64 |
|  | 50 | 49.12±1.01 | 2.07 | -1.77 | 49.34±1.00 | 2.03 | -1.32 |
|  | 80 | 79.20±1.70 | 2.14 | -1.00 | 79.29±1.50 | 1.89 | -0.89 |
| Bavachinin | 2.5 | 2.47±0.03 | 1.27 | -1.07 | 2.48±0.02 | 0.86 | -0.78 |
|  | 25 | 24.72±0.45 | 1.82 | -1.13 | 24.72±0.39 | 1.57 | -1.13 |
|  | 40 | 38.75±1.26 | 3.26 | -3.13 | 38.79±1.31 | 3.37 | -3.03 |

**Table 7.** Intra-assay and inter-assay precision, accuracy of 12 compounds in rat testes (*n* = 6).

| Compound | Concentrations  (ng/mL) |  | Intraday |  |  | Interday |  |
| --- | --- | --- | --- | --- | --- | --- | --- |
|  |  | Measured  (ng/mL) | Precision(%) | Accuracy(%) | Measured  (ng/mL) | Precision (%) | Accuracy  (%) |
| Psoralen | 2.5 | 2.37±0.08 | 3.27 | -5.11 | 2.36±0.10 | 4.04 | -5.46 |
|  | 500 | 481.07±18.08 | 3.76 | -3.79 | 454.62±32.04 | 7.05 | -9.08 |
|  | 800 | 753.66±42.07 | 5.58 | -5.79 | 754.10±33.27 | 4.41 | -5.74 |
| Isopsoralen | 12.5 | 12.24±0.29 | 2.35 | -2.12 | 12.16±0.48 | 3.97 | -2.69 |
|  | 500 | 471.50±15.76 | 3.34 | -5.70 | 476.99±13.46 | 2.82 | -4.60 |
|  | 800 | 750.42±36.59 | 4.88 | -6.20 | 762.81±39.49 | 5.18 | -4.65 |
| Psoralenoside | 12.5 | 12.18±0.39 | 3.17 | -2.58 | 11.99±0.51 | 4.24 | -4.07 |
|  | 500 | 486.08±11.02 | 2.27 | -2.78 | 461.49±28.53 | 6.18 | -7.70 |
|  | 800 | 771.67±29.50 | 3.82 | -3.54 | 764.69±33.40 | 4.37 | -4.41 |
| Bavachin | 2.5 | 2.41±0.09 | 3.70 | -3.42 | 2.36±0.10 | 4.18 | -5.56 |
|  | 25 | 23.75±1.16 | 4.87 | -5.01 | 23.57±1.06 | 4.48 | -5.73 |
|  | 40 | 37.87±2.97 | 7.85 | -5.32 | 37.28±2.57 | 6.90 | -6.81 |
| Isobavachin | 1.25 | 1.20±0.03 | 2.70 | -4.02 | 1.20±0.04 | 3.08 | -4.22 |
|  | 25 | 23.60±1.48 | 6.26 | -5.62 | 22.72±1.92 | 8.46 | -9.13 |
|  | 40 | 37.52±3.32 | 8.84 | -6.20 | 36.94±3.03 | 8.21 | -7.65 |
| Bavachalcone | 1.25 | 1.19±0.04 | 3.05 | -5.19 | 1.19±0.03 | 2.85 | -4.65 |
|  | 25 | 23.60±1.26 | 5.33 | -5.59 | 23.79±1.46 | 6.14 | -4.85 |
|  | 40 | 37.50±1.39 | 3.71 | -6.25 | 36.98±2.04 | 5.51 | -7.56 |
| Isobavachalcone | 1.25 | 1.21±0.03 | 2.19 | -3.58 | 1.21±0.04 | 3.01 | -3.29 |
|  | 25 | 24.05±1.03 | 4.30 | -3.79 | 23.78±1.19 | 4.99 | -4.87 |
|  | 40 | 38.10±2.04 | 5.36 | -4.76 | 37.55±2.36 | 6.27 | -6.12 |
| Pinoresinol diglucoside | 1.25 | 1.21±0.03 | 2.42 | -3.12 | 1.20±0.05 | 3.87 | -3.76 |
|  | 25 | 24.12±1.04 | 4.30 | -3.53 | 23.75±1.57 | 6.61 | -4.99 |
|  | 40 | 38.19±1.85 | 4.84 | -4.53 | 37.80±1.89 | 4.99 | -5.49 |
| Geniposidic acid | 2.5 | 2.37±0.03 | 1.11 | -5.13 | 2.38±0.08 | 3.49 | -4.75 |
|  | 125 | 118.55±5.34 | 4.50 | -5.16 | 118.92±5.80 | 4.87 | -4.86 |
|  | 200 | 185.98±13.50 | 7.26 | -7.01 | 186.59±11.42 | 6.12 | -6.70 |
| Psoralidin | 1.25 | 1.22±0.02 | 1.99 | -2.64 | 1.20±0.06 | 5.11 | -4.25 |
|  | 25 | 23.87±1.44 | 6.03 | -4.52 | 23.35±1.65 | 7.06 | -6.60 |
|  | 40 | 38.04±1.87 | 4.92 | -4.90 | 37.98±1.95 | 5.14 | -5.05 |
| Neobavaisoflavone | 2.5 | 2.45±0.12 | 4.89 | -1.93 | 2.47±0.08 | 3.34 | -1.23 |
|  | 25 | 24.25±0.82 | 3.37 | -3.00 | 24.67±1.85 | 7.48 | -1.31 |
|  | 40 | 38.14±2.45 | 6.43 | -4.66 | 38.74±3.58 | 9.24 | -3.15 |
| Bavachinin | 2.5 | 2.39±0.08 | 3.49 | -4.33 | 2.40±0.10 | 4.20 | -3.93 |
|  | 50 | 47.79±5.92 | 12.40 | -4.43 | 47.78±3.95 | 8.28 | -4.43 |
|  | 80 | 76.23±4.64 | 6.09 | -4.72 | 76.35±6.75 | 8.84 | -4.57 |

**Extract Recovery and Matrix Effect**

The extract recoveries and matrix effect in the heart, liver, spleen, lung, uterus, ovary, and testes are shown in Table 8–14. The results indicated that the extraction method was suitable for the treatment of the heart, liver, spleen, lung, uterus, ovary, and testis tissue samples.

**Table 8.** The extract recoveries and matrix effect of 12 compounds in rat heart (*n* = 6).

| Compound | Concentrations(ng/mL) | Extract recovery (%) | RSD (%) | Matrix effect (%) | RSD (%) |
| --- | --- | --- | --- | --- | --- |
| Psoralen | 2.5 | 77.81±3.22 | 4.14 | 87.57±4.38 | 5.01 |
|  | 500 | 85.71±5.40 | 6.30 | 95.30±8.17 | 8.58 |
|  | 800 | 84.23±7.11 | 8.44 | 94.56±8.92 | 9.44 |
| Isopsoralen | 2.5 | 85.50±2.92 | 3.41 | 94.04±7.69 | 8.18 |
|  | 500 | 88.51±3.68 | 4.16 | 99.15±8.12 | 8.19 |
|  | 800 | 80.28±7.00 | 8.72 | 90.56±11.49 | 12.68 |
| Psoralenoside | 12.5 | 80.11±1.46 | 1.83 | 90.34±5.85 | 6.48 |
|  | 500 | 75.12±3.37 | 4.49 | 85.49±7.65 | 8.95 |
|  | 800 | 78.47±4.71 | 6.00 | 88.04±8.07 | 9.17 |
| Bavachin | 1 | 84.07±8.97 | 10.66 | 81.49±5.21 | 6.40 |
|  | 25 | 82.48±6.98 | 8.46 | 93.97±9.11 | 9.69 |
|  | 40 | 80.55±9.71 | 12.05 | 91.70±11.08 | 12.08 |
| Isobavachin | 1 | 80.13±5.38 | 6.71 | 82.15±3.16 | 3.84 |
|  | 25 | 80.16±4.40 | 5.49 | 90.54±6.99 | 7.72 |
|  | 40 | 82.23±5.11 | 6.22 | 84.03±6.76 | 8.05 |
| Bavachalcone | 1 | 81.23±6.28 | 7.73 | 84.12±2.96 | 3.52 |
|  | 12.5 | 75.49±3.53 | 4.68 | 85.29±4.24 | 4.98 |
|  | 20 | 77.35±5.51 | 7.13 | 87.15±7.10 | 8.15 |
| Isobavachalcone | 2.5 | 81.79±1.13 | 1.38 | 91.05±3.19 | 3.50 |
|  | 25 | 77.37±4.51 | 5.83 | 87.49±3.53 | 4.04 |
|  | 40 | 83.98±7.15 | 8.51 | 92.40±5.77 | 6.25 |
| Pinoresinol diglucoside | 1.25 | 79.15±3.92 | 4.95 | 89.88±3.51 | 3.90 |
|  | 50 | 82.99±7.53 | 9.08 | 83.40±4.04 | 4.84 |
|  | 80 | 82.45±6.73 | 8.16 | 93.06±6.93 | 7.44 |
| Geniposidic acid | 2.5 | 82.47±7.50 | 9.10 | 81.78±4.34 | 5.31 |
|  | 250 | 82.36±4.52 | 5.49 | 92.19±7.83 | 8.49 |
|  | 400 | 83.22±5.89 | 7.08 | 94.19±8.51 | 9.04 |
| Psoralidin | 2.5 | 80.90±4.04 | 4.99 | 81.70±3.09 | 3.78 |
|  | 125 | 85.24±3.91 | 4.59 | 95.83±5.62 | 5.87 |
|  | 200 | 84.76±5.20 | 6.13 | 93.50±8.61 | 9.21 |
| Neobavaisoflavone | 1.25 | 78.85±4.51 | 5.72 | 88.07±6.24 | 7.08 |
|  | 25 | 80.52±7.98 | 9.92 | 83.42±6.62 | 7.94 |
|  | 40 | 75.92±7.25 | 9.55 | 86.13±7.16 | 8.32 |
| Bavachinin | 2.5 | 83.66±1.75 | 2.09 | 94.60±4.75 | 5.02 |
|  | 50 | 87.90±6.66 | 7.57 | 83.29±6.08 | 7.30 |
|  | 80 | 75.06±7.33 | 9.76 | 85.69±8.05 | 9.40 |

**Table 9. The extraction recoveries and matrix effect of 12 compounds in rat liver (*n* = 6).**

| Compound | Concentrations(ng/mL) | Extract recovery (%) | RSD (%) | Matrix effect (%) | RSD (%) |
| --- | --- | --- | --- | --- | --- |
| Psoralen | 12.5 | 83.28±1.24 | 1.49 | 93.00±3.08 | 3.31 |
|  | 500 | 84.30±1.49 | 1.76 | 94.82±5.66 | 5.97 |
|  | 800 | 85.75±2.54 | 2.97 | 95.62±5.90 | 6.17 |
| Isopsoralen | 12.5 | 81.86±1.32 | 1.62 | 92.53±3.17 | 3.43 |
|  | 500 | 86.58±1.50 | 1.73 | 98.24±4.69 | 4.77 |
|  | 800 | 85.83±2.53 | 2.95 | 95.54±5.92 | 6.19 |
| Psoralenoside | 12.5 | 82.62±1.27 | 1.54 | 92.84±3.04 | 3.27 |
|  | 500 | 88.07±2.11 | 2.40 | 98.92±3.31 | 3.35 |
|  | 800 | 87.49±2.50 | 2.86 | 96.23±6.15 | 6.39 |
| Bavachin | 2.5 | 84.48±2.98 | 3.53 | 94.33±5.14 | 5.45 |
|  | 250 | 86.16±9.36 | 10.86 | 96.52±5.76 | 5.97 |
|  | 400 | 88.96±2.66 | 2.99 | 98.91±5.69 | 5.75 |
| Isobavachin | 2.5 | 80.31±2.80 | 3.48 | 90.93±5.81 | 6.38 |
|  | 125 | 85.71±6.07 | 7.08 | 95.03±3.78 | 3.98 |
|  | 200 | 76.13±4.31 | 5.67 | 87.21±7.55 | 8.66 |
| Bavachalcone | 1.25 | 88.01±0.46 | 0.52 | 98.07±0.95 | 0.97 |
|  | 125 | 87.72±2.21 | 2.52 | 97.47±5.20 | 5.33 |
|  | 200 | 79.44±4.20 | 5.29 | 90.22±7.35 | 8.14 |
| Isobavachalcone | 2.5 | 87.78±5.24 | 5.97 | 97.43±2.77 | 2.85 |
|  | 50 | 84.78±8.69 | 10.25 | 94.88±6.49 | 6.84 |
|  | 80 | 82.12±3.68 | 4.48 | 92.33±4.98 | 5.39 |
| Pinoresinol diglucoside | 1.25 | 81.43±6.93 | 8.51 | 91.02±3.51 | 3.86 |
|  | 125 | 87.72±1.73 | 1.97 | 97.43±5.18 | 5.31 |
|  | 200 | 82.30±1.87 | 2.27 | 92.63±6.14 | 6.63 |
| Geniposidic acid | 2.5 | 87.80±5.26 | 6.00 | 97.43±2.77 | 2.85 |
|  | 500 | 85.46±8.82 | 10.32 | 95.03±6.57 | 6.92 |
|  | 800 | 81.96±3.43 | 4.18 | 92.23±5.21 | 5.65 |
| Psoralidin | 2.5 | 84.70±2.97 | 3.50 | 84.70±2.97 | 3.50 |
|  | 250 | 86.16±6.96 | 8.08 | 96.52±5.76 | 5.97 |
|  | 400 | 88.95±2.66 | 2.99 | 98.91±5.69 | 5.75 |
| Neobavaisoflavone | 2.5 | 84.71±5.32 | 6.28 | 94.52±3.27 | 3.46 |
|  | 500 | 85.46±7.47 | 8.74 | 95.02±6.57 | 6.92 |
|  | 800 | 95.02±6.57 | 6.92 | 92.29±5.29 | 5.73 |
| Bavachinin | 2.5 | 84.86±5.32 | 6.27 | 94.74±2.79 | 2.94 |
|  | 500 | 85.49±7.51 | 8.79 | 95.02±6.57 | 6.91 |
|  | 800 | 82.24±3.62 | 4.40 | 92.28±5.28 | 5.72 |

**Table 10. The extraction recoveries and matrix effect of 12 compounds in rat spleen (*n* = 6).**

| Compound | Concentrations(ng/mL) | Extract recovery (%) | RSD (%) | Matrix effect (%) | RSD (%) |
| --- | --- | --- | --- | --- | --- |
| Psoralen | 12.5 | 84.55±3.84 | 4.54 | 94.16±6.53 | 6.93 |
|  | 500 | 87.96±11.90 | 13.53 | 97.27±2.89 | 2.97 |
|  | 800 | 84.44±1.71 | 2.02 | 94.08±4.86 | 5.17 |
| Isopsoralen | 12.5 | 84.15±7.02 | 8.35 | 94.42±8.23 | 8.72 |
|  | 500 | 84.78±10.88 | 12.83 | 94.74±7.91 | 8.35 |
|  | 800 | 83.19±1.93 | 2.32 | 93.37±7.18 | 7.69 |
| Psoralenoside | 12.5 | 81.43±5.86 | 7.20 | 91.60±8.53 | 9.31 |
|  | 500 | 84.34±8.72 | 10.34 | 94.22±7.52 | 7.98 |
|  | 800 | 83.19±1.93 | 2.32 | 93.37±7.18 | 7.69 |
| Bavachin | 2.5 | 83.25±6.70 | 8.05 | 93.95±10.33 | 11.00 |
|  | 100 | 81.19±4.98 | 6.14 | 91.36±7.89 | 8.63 |
|  | 160 | 82.32±4.93 | 5.99 | 92.10±7.71 | 8.37 |
| Isobavachin | 1.25 | 80.70±7.88 | 9.77 | 90.03±9.43 | 10.47 |
|  | 50 | 87.55±5.82 | 6.65 | 97.28±3.01 | 3.09 |
|  | 80 | 86.92±3.02 | 3.48 | 96.70±6.45 | 6.67 |
| Bavachalcone | 1.25 | 85.16±6.18 | 7.26 | 95.92±9.15 | 9.54 |
|  | 25 | 81.95±3.54 | 4.32 | 91.06±4.32 | 4.75 |
|  | 40 | 84.36±8.54 | 10.13 | 94.36±8.48 | 8.98 |
| Isobavachalcone | 1.25 | 85.04±7.42 | 8.73 | 95.22±7.72 | 8.11 |
|  | 125 | 85.20±2.64 | 3.09 | 95.60±3.34 | 3.49 |
|  | 200 | 87.76±6.00 | 6.83 | 97.27±4.87 | 5.01 |
| Pinoresinol diglucoside | 1.25 | 87.02±7.50 | 8.62 | 97.91±3.90 | 3.99 |
|  | 50 | 86.08±7.48 | 8.69 | 96.91±3.05 | 3.15 |
|  | 80 | 84.70±1.49 | 1.76 | 94.70±7.08 | 7.47 |
| Geniposidic acid | 2.5 | 88.16±4.08 | 4.62 | 98.16±2.89 | 2.95 |
|  | 250 | 81.29±3.43 | 4.21 | 91.25±3.25 | 3.57 |
|  | 400 | 83.29±1.99 | 2.39 | 93.76±8.41 | 8.97 |
| Psoralidin | 2.5 | 86.90±1.78 | 2.05 | 97.49±3.17 | 3.25 |
|  | 250 | 83.39±1.26 | 1.51 | 93.13±1.95 | 2.10 |
|  | 400 | 84.39±1.35 | 1.60 | 94.24±8.34 | 8.85 |
| Neobavaisoflavone | 2.5 | 85.48±1.62 | 1.90 | 95.01±3.49 | 3.67 |
|  | 250 | 83.62±1.11 | 1.32 | 93.24±2.41 | 2.58 |
|  | 400 | 85.21±1.48 | 1.74 | 95.25±7.51 | 7.88 |
| Bavachinin | 2.5 | 83.17±4.14 | 4.98 | 93.44±4.57 | 4.89 |
|  | 500 | 83.55±6.82 | 8.16 | 93.20±4.02 | 4.31 |
|  | 800 | 84.44±1.71 | 2.02 | 94.08±4.86 | 5.17 |

**Table 11.** The extraction recoveries and matrix effect of 12 compounds in rat lung (*n* = 6).

| Compound | Concentrations(ng/mL) | Extract recovery (%) | RSD (%) | Matrix effect (%) | RSD (%) |
| --- | --- | --- | --- | --- | --- |
| Psoralen | 12.5 | 87.42±2.71 | 3.10 | 93.77±1.84 | 1.96 |
|  | 500 | 90.78±1.76 | 1.94 | 91.69±1.47 | 1.60 |
|  | 800 | 89.44±2.99 | 3.35 | 96.70±2.39 | 2.48 |
| Isopsoralen | 12.5 | 88.63±5.64 | 6.36 | 90.33±3.71 | 4.11 |
|  | 500 | 84.48±2.47 | 2.93 | 89.00±1.49 | 1.68 |
|  | 800 | 88.13±2.53 | 2.87 | 95.51±3.83 | 4.01 |
| Psoralenoside | 2.5 | 84.91±2.74 | 3.23 | 94.76±5.41 | 5.71 |
|  | 1000 | 80.80±1.50 | 1.86 | 90.36±5.22 | 5.77 |
|  | 1600 | 90.00±3.03 | 3.36 | 98.33±2.83 | 2.88 |
| Bavachin | 1.25 | 89.78±4.29 | 4.78 | 93.66±3.11 | 3.32 |
|  | 100 | 81.08±1.64 | 2.03 | 90.89±4.72 | 5.19 |
|  | 160 | 88.83±3.27 | 3.68 | 97.97±2.98 | 3.04 |
| Isobavachin | 1.25 | 88.34±4.00 | 4.53 | 92.78±4.65 | 5.01 |
|  | 50 | 82.34±2.88 | 3.49 | 90.10±3.05 | 3.38 |
|  | 80 | 89.23±1.63 | 1.82 | 94.75±1.80 | 1.89 |
| Bavachalcone | 1.25 | 90.37±2.47 | 2.74 | 93.84±4.44 | 4.73 |
|  | 50 | 84.76±1.85 | 2.18 | 95.14±2.09 | 2.20 |
|  | 80 | 89.07±2.22 | 2.50 | 97.70±1.62 | 1.65 |
| Isobavachalcone | 2.5 | 89.72±8.92 | 9.94 | 97.97±4.23 | 4.32 |
|  | 12.5 | 82.12±1.55 | 1.89 | 89.73±3.51 | 3.91 |
|  | 200 | 89.36±2.68 | 3.00 | 97.51±4.83 | 4.95 |
| Pinoresinol diglucoside | 1.25 | 82.53±4.04 | 4.90 | 87.91±2.70 | 3.08 |
|  | 250 | 77.72±2.72 | 3.50 | 86.85±6.04 | 6.96 |
|  | 400 | 90.87±5.60 | 6.17 | 98.33±2.23 | 2.27 |
| Geniposidic acid | 2.5 | 88.75±6.58 | 7.42 | 97.99±3.86 | 3.94 |
|  | 500 | 85.03±2.49 | 2.93 | 98.68±2.69 | 2.73 |
|  | 800 | 83.48±1.96 | 2.35 | 90.16±2.76 | 3.06 |
| Psoralidin | 2.5 | 85.73±3.64 | 4.25 | 93.64±2.86 | 3.05 |
|  | 250 | 90.10±1.42 | 1.58 | 92.57±2.51 | 2.71 |
|  | 400 | 88.63±1.38 | 1.56 | 93.05±2.48 | 2.67 |
| Neobavaisoflavone | 2.5 | 82.11±4.79 | 5.83 | 91.59±1.85 | 2.01 |
|  | 150 | 84.98±2.49 | 2.92 | 90.65±3.49 | 3.85 |
|  | 240 | 87.46±1.16 | 1.33 | 93.08±2.99 | 3.21 |
| Bavachinin | 2.5 | 85.51±2.72 | 3.19 | 95.55±2.07 | 2.17 |
|  | 250 | 84.16±1.63 | 1.94 | 85.01±1.36 | 1.60 |
|  | 400 | 93.63±3.13 | 3.35 | 98.46±1.74 | 1.76 |

**Table 12. The extraction recoveries and matrix effect of 12 compounds in rat uterus (*n* = 6).**

| Compound | Concentrations(ng/mL) | Extract recovery (%) | RSD (%) | Matrix effect (%) | RSD (%) |
| --- | --- | --- | --- | --- | --- |
| Psoralen | 2.5 | 85.21±6.01 | 7.06 | 91.42±4.46 | 4.88 |
|  | 500 | 89.35±1.51 | 1.69 | 94.83±1.29 | 1.36 |
|  | 800 | 88.77±3.25 | 3.66 | 95.34±2.09 | 2.20 |
| Isopsoralen | 2.5 | 84.29±2.97 | 3.53 | 92.53±2.10 | 2.27 |
|  | 250 | 83.78±2.54 | 3.03 | 94.47±11.48 | 12.15 |
|  | 400 | 82.15±4.29 | 5.22 | 91.73±2.84 | 3.09 |
| Psoralenoside | 12.5 | 84.97±2.36 | 2.78 | 93.31±2.04 | 2.19 |
|  | 1000 | 82.14±3.14 | 3.82 | 93.33±5.38 | 5.77 |
|  | 1600 | 83.48±0.66 | 0.79 | 94.04±3.44 | 3.66 |
| Bavachin | 2.5 | 84.02±2.79 | 3.32 | 91.80±3.68 | 4.01 |
|  | 100 | 85.78±3.14 | 3.66 | 92.36±3.09 | 3.34 |
|  | 160 | 83.82±1.64 | 1.96 | 92.01±2.07 | 2.25 |
| Isobavachin | 0.25 | 81.37±4.36 | 5.35 | 94.41±3.04 | 3.22 |
|  | 25 | 81.98±1.72 | 2.10 | 93.01±1.39 | 1.49 |
|  | 40 | 83.37±1.47 | 1.77 | 92.05±1.52 | 1.65 |
| Bavachalcone | 1.25 | 86.63±1.60 | 1.85 | 92.20±1.65 | 1.79 |
|  | 125 | 82.68±1.75 | 2.12 | 94.02±1.50 | 1.60 |
|  | 200 | 84.56±1.69 | 2.00 | 92.22±2.07 | 2.24 |
| Isobavachalcone | 2.5 | 83.29±1.57 | 1.89 | 94.32±3.58 | 3.80 |
|  | 25 | 80.82±0.86 | 1.06 | 93.38±2.95 | 3.16 |
|  | 40 | 85.11±2.25 | 2.64 | 92.52±1.08 | 1.17 |
| Pinoresinol diglucoside | 1.25 | 88.46±1.68 | 1.90 | 94.94±1.10 | 1.16 |
|  | 50 | 87.00±0.87 | 1.00 | 93.5±1.75 | 1.87 |
|  | 80 | 88.59±1.12 | 1.27 | 94.82±0.83 | 0.88 |
| Geniposidic acid | 1.25 | 84.40±2.01 | 2.38 | 93.87±5.49 | 5.85 |
|  | 250 | 83.45±3.36 | 4.03 | 92.71±2.46 | 2.65 |
|  | 400 | 85.46±1.58 | 1.84 | 93.16±1.93 | 2.07 |
| Psoralidin | 2.5 | 82.87±4.13 | 4.98 | 93.58±1.54 | 1.64 |
|  | 125 | 81.71±1.81 | 2.21 | 89.76±2.66 | 2.96 |
|  | 200 | 79.57±0.84 | 1.06 | 91.99±1.98 | 2.15 |
| Neobavaisoflavone | 2.5 | 86.79±1.29 | 1.49 | 95.47±4.69 | 4.91 |
|  | 25 | 83.51±3.36 | 4.02 | 93.81±1.86 | 1.98 |
|  | 40 | 83.08±2.81 | 3.38 | 92.81±2.42 | 2.61 |
| Bavachinin | 2.5 | 87.86±1.04 | 1.18 | 92.85±1.45 | 1.57 |
|  | 25 | 86.79±1.53 | 1.76 | 94.39±1.59 | 1.69 |
|  | 40 | 87.60±0.61 | 0.69 | 94.82±0.96 | 1.01 |

**Table 13. The extraction recoveries and matrix effect of 12 compounds in rat ovary (*n* = 6).**

| Compound | Concentrations(ng/mL) | Extract recovery (%) | RSD (%) | Matrix effect (%) | RSD (%) |
| --- | --- | --- | --- | --- | --- |
| Psoralen | 12.5 | 86.94±3.04 | 3.50 | 92.79±1.54 | 1.66 |
|  | 500 | 88.40±0.85 | 0.96 | 92.47±1.05 | 1.13 |
|  | 800 | 87.69±0.94 | 1.07 | 93.55±1.49 | 1.59 |
| Isopsoralen | 12.5 | 86.99±1.18 | 1.35 | 94.10±0.75 | 0.80 |
|  | 500 | 87.82±0.51 | 0.59 | 94.11±1.08 | 1.15 |
|  | 800 | 88.20±0.66 | 0.75 | 94.93±0.73 | 0.77 |
| Psoralenoside | 12.5 | 87.40±1.44 | 1.65 | 92.73±2.50 | 2.70 |
|  | 1000 | 84.14±2.85 | 3.39 | 93.37±2.00 | 2.14 |
|  | 1600 | 84.60±1.36 | 1.61 | 94.51±2.34 | 2.48 |
| Bavachin | 2.5 | 84.65±0.58 | 0.69 | 93.54±1.45 | 1.55 |
|  | 25 | 85.39±1.13 | 1.32 | 93.76±0.89 | 0.95 |
|  | 40 | 87.32±1.51 | 1.73 | 92.55±1.34 | 1.44 |
| Isobavachin | 2.5 | 87.90±1.03 | 1.17 | 94.47±1.50 | 1.59 |
|  | 25 | 87.69±1.34 | 1.53 | 94.10±1.57 | 1.67 |
|  | 40 | 87.58±0.54 | 0.61 | 94.09±1.56 | 1.66 |
| Bavachalcone | 1.25 | 82.50±1.85 | 2.24 | 92.24±1.61 | 1.75 |
|  | 25 | 84.73±1.72 | 2.03 | 94.46±1.08 | 1.15 |
|  | 40 | 85.56±1.39 | 1.62 | 94.22±1.35 | 1.43 |
| Isobavachalcone | 1.25 | 85.19±1.28 | 1.51 | 94.10±0.81 | 0.86 |
|  | 25 | 85.79±0.80 | 0.94 | 93.51±0.83 | 0.89 |
|  | 40 | 86.60±0.98 | 1.14 | 94.04±0.60 | 0.64 |
| Pinoresinol diglucoside | 1.25 | 84.88±1.99 | 2.35 | 94.03±1.46 | 1.55 |
|  | 50 | 86.16±2.96 | 3.44 | 92.25±2.24 | 2.43 |
|  | 80 | 87.11±0.94 | 1.08 | 93.45±1.23 | 1.31 |
| Geniposidic acid | 2.5 | 87.35±0.93 | 1.06 | 93.11±1.18 | 1.27 |
|  | 500 | 85.27±1.19 | 1.39 | 92.86±0.84 | 0.90 |
|  | 800 | 86.21±0.92 | 1.06 | 93.60±0.91 | 0.97 |
| Psoralidin | 1.25 | 86.10±2.24 | 2.61 | 93.64±1.34 | 1.43 |
|  | 50 | 87.17±1.72 | 1.98 | 93.38±1.29 | 1.38 |
|  | 80 | 86.75±0.60 | 0.70 | 93.82±0.52 | 0.56 |
| Neobavaisoflavone | 2.5 | 83.90±2.17 | 2.59 | 93.72±1.50 | 1.60 |
|  | 50 | 83.02±2.87 | 3.46 | 92.77±0.96 | 1.04 |
|  | 80 | 88.78±1.91 | 2.15 | 93.94±1.45 | 1.54 |
| Bavachinin | 2.5 | 86.49±2.39 | 2.77 | 93.01±1.18 | 1.27 |
|  | 25 | 86.38±1.66 | 1.93 | 92.81±1.09 | 1.18 |
|  | 40 | 84.53±1.32 | 1.57 | 92.10±3.22 | 3.50 |

**Table 14. The extraction recoveries and matrix effect of 12 compounds in rat testes (*n* = 6).**

| Compound | Concentrations(ng/mL) | Extract recovery (%) | RSD (%) | Matrix effect (%) | RSD (%) |
| --- | --- | --- | --- | --- | --- |
| Psoralen | 2.5 | 85.86±3.90 | 4.54 | 94.76±4.82 | 5.08 |
|  | 500 | 89.95±1.49 | 1.66 | 99.93±4.44 | 4.45 |
|  | 800 | 89.09±4.93 | 5.53 | 98.21±3.47 | 3.53 |
| Isopsoralen | 12.5 | 89.45±2.95 | 3.30 | 97.20±2.30 | 2.37 |
|  | 500 | 89.63±2.20 | 2.45 | 99.30±2.75 | 2.77 |
|  | 800 | 89.14±1.85 | 2.08 | 98.74±1.56 | 1.58 |
| Psoralenoside | 12.5 | 89.68±3.69 | 4.12 | 98.60±4.38 | 4.44 |
|  | 500 | 88.52±5.26 | 5.95 | 96.52±5.04 | 5.22 |
|  | 800 | 89.47±4.45 | 4.98 | 97.74±3.72 | 3.80 |
| Bavachin | 2.5 | 84.82±2.04 | 2.41 | 94.30±3.53 | 3.74 |
|  | 25 | 84.98±2.83 | 3.32 | 95.69±3.95 | 4.13 |
|  | 40 | 81.63±3.29 | 4.03 | 91.27±4.10 | 4.49 |
| Isobavachin | 1.25 | 81.97±3.87 | 4.73 | 91.67±4.30 | 4.69 |
|  | 25 | 84.40±4.21 | 4.99 | 93.93±5.24 | 5.57 |
|  | 40 | 83.85±4.40 | 5.25 | 93.76±7.56 | 8.06 |
| Bavachalcone | 1.25 | 84.72±4.04 | 4.76 | 94.65±4.61 | 4.88 |
|  | 25 | 84.64±4.35 | 5.14 | 94.91±5.43 | 5.72 |
|  | 40 | 83.97±4.45 | 5.30 | 94.07±4.59 | 4.88 |
| Isobavachalcone | 1.25 | 82.80±4.22 | 5.09 | 92.10±4.41 | 4.78 |
|  | 25 | 84.17±5.56 | 6.60 | 95.73±4.88 | 5.10 |
|  | 40 | 84.68±5.76 | 6.80 | 93.65±5.03 | 5.37 |
| Pinoresinol diglucoside | 1.25 | 84.90±3.73 | 4.40 | 94.13±5.19 | 5.52 |
|  | 25 | 83.55±5.48 | 6.56 | 93.74±7.17 | 7.65 |
|  | 40 | 83.73±7.45 | 8.89 | 92.38±7.41 | 8.02 |
| Geniposidic acid | 2.5 | 84.78±1.38 | 1.62 | 93.94±4.93 | 5.25 |
|  | 125 | 84.49±3.64 | 4.31 | 94.28±4.98 | 5.28 |
|  | 200 | 79.32±4.66 | 5.88 | 89.36±5.75 | 6.44 |
| Psoralidin | 1.25 | 88.15±6.55 | 7.43 | 98.94±4.62 | 4.67 |
|  | 25 | 89.95±6.02 | 6.70 | 98.16±5.55 | 5.65 |
|  | 40 | 89.51±6.62 | 7.39 | 98.41±2.15 | 2.18 |
| Neobavaisoflavone | 2.5 | 84.89±5.07 | 5.97 | 94.32±8.20 | 8.69 |
|  | 25 | 84.12±6.80 | 8.09 | 94.86±8.72 | 9.19 |
|  | 40 | 79.33±7.83 | 9.87 | 89.34±8.37 | 9.37 |
| Bavachinin | 2.5 | 84.02±4.68 | 5.57 | 92.84±2.05 | 2.21 |
|  | 50 | 84.24±5.24 | 6.23 | 92.72±5.01 | 5.40 |
|  | 80 | 83.96±5.65 | 6.73 | 92.15±5.72 | 6.21 |

**Stability**

The results of compound stability in the heart, liver, spleen, lung, uterus, ovary, and testis tissue under diﬀerent storage conditions are presented in Table 15–21. Results showed that samples were all stable, and the storage conditions did not affect the determination of the chemical components in the experimental tissue samples.

**Table 15. The stability of 12 compounds in rat heart (*n* = 6).**

| Compound | Concentrations  (ng/mL) | Freeze-thaw stability | | Short-term stability | | Long-term stability | | Pre-injection stability | |
| --- | --- | --- | --- | --- | --- | --- | --- | --- | --- |
|  |  | Measured  (ng/mL) | RE  (%) | Measured  (ng/mL) | RE  (%) | Measured  (ng/mL) | RE  (%) | Measured  (ng/mL) | RE  (%) |
| Psoralen | 2.5 | 2.44±0.02 | -2.36 | 2.42±0.03 | -3.11 | 2.39±0.12 | -4.40 | 2.42±0.09 | -3.06 |
|  | 500 | 482.97±33.50 | -3.41 | 478.50±34.95 | -4.30 | 473.78±3.90 | -5.24 | 474.63±18.90 | -5.07 |
|  | 800 | 759.92±41.09 | -5.01 | 740.87±11.26 | -7.39 | 741.77±39.23 | -7.28 | 747.97±26.19 | -6.50 |
| Isopsoralen | 2.5 | 2.36±0.10 | -5.71 | 2.41±0.03 | -3.56 | 2.42±0.07 | -3.18 | 2.42±0.03 | -3.32 |
|  | 500 | 462.02±11.23 | -7.60 | 476.57±2.58 | -4.69 | 469.38±6.25 | -6.12 | 479.55±28.73 | -4.09 |
|  | 800 | 734.88±26.59 | -8.14 | 754.18±2.72 | -5.73 | 719.83±16.18 | -10.02 | 724.20±24.31 | -9.48 |
| Psoralenoside | 12.5 | 12.22±0.10 | -2.25 | 12.18±0.10 | -2.56 | 12.03±0.51 | -3.75 | 11.95±0.17 | -4.39 |
|  | 500 | 479.60±10.42 | -4.08 | 473.77±2.54 | -5.25 | 471.22±18.94 | -5.76 | 473.03±7.84 | -5.39 |
|  | 800 | 745.33±31.73 | -6.83 | 732.67±6.41 | -8.42 | 738.17±14.19 | -7.73 | 725.50±32.57 | -9.31 |
| Bavachin | 1 | 0.95±0.02 | -4.70 | 0.93±0.03 | -6.54 | 0.95±0.03 | -4.92 | 0.93±0.03 | -6.60 |
|  | 25 | 23.40±0.22 | -6.41 | 22.61±1.27 | -9.57 | 23.33±1.35 | -6.69 | 22.59±2.10 | -9.64 |
|  | 40 | 35.37±0.78 | -11.57 | 35.64±1.63 | -10.91 | 36.20±2.42 | -9.50 | 35.73±3.25 | -10.69 |
| Isobavachin | 1 | 0.97±0.02 | -3.46 | 0.96±0.03 | -4.04 | 0.97±0.02 | -3.50 | 0.93±0.03 | -6.65 |
|  | 25 | 24.07±0.52 | -3.72 | 23.47±0.88 | -6.13 | 23.78±0.58 | -4.90 | 22.48±0.89 | -10.09 |
|  | 40 | 37.00±1.80 | -7.50 | 35.64±0.28 | -10.90 | 36.01±1.52 | -9.97 | 34.15±0.49 | -14.62 |
| Bavachalcone | 1 | 0.96±0.03 | -4.48 | 0.96±0.02 | -3.90 | 0.95±0.04 | -4.83 | 0.95±0.01 | -5.24 |
|  | 12.5 | 11.79±0.14 | -5.68 | 11.96±0.35 | -4.29 | 11.79±0.34 | -5.69 | 11.67±0.22 | -6.67 |
|  | 20 | 18.86±0.55 | -5.71 | 18.71±0.95 | -6.46 | 18.77±0.45 | -6.14 | 18.36±0.92 | -8.23 |
| Isobavachalcone | 2.5 | 2.42±0.06 | -3.12 | 2.38±0.07 | -4.91 | 2.41±0.03 | -3.46 | 2.46±0.03 | -1.76 |
|  | 25 | 23.90±0.69 | -4.40 | 22.86±0.99 | -8.57 | 23.38±0.89 | -6.48 | 24.17±0.59 | -3.34 |
|  | 40 | 36.59±1.59 | -8.53 | 36.05±2.29 | -9.87 | 36.88±0.57 | -7.79 | 37.32±0.71 | -6.70 |
| Pinoresinol diglucoside | 1.25 | 1.20±0.07 | -4.26 | 1.23±0.03 | -1.96 | 1.22±0.02 | -2.64 | 1.21±0.02 | -3.19 |
|  | 50 | 45.73±1.19 | -8.53 | 47.03±1.18 | -5.95 | 46.88±1.73 | -6.24 | 47.83±0.74 | -4.34 |
|  | 80 | 69.75±1.21 | -12.81 | 72.22±2.27 | -9.72 | 73.38±2.29 | -8.27 | 74.66±3.65 | -6.68 |
| Geniposidic acid | 2.5 | 2.42±0.06 | -3.12 | 2.38±0.07 | -4.91 | 2.41±0.03 | -3.46 | 2.46±0.03 | -1.76 |
|  | 250 | 235.40±5.59 | -5.84 | 225.36±8.49 | -9.86 | 230.38±8.91 | -7.85 | 238.33±3.75 | -4.67 |
|  | 400 | 360.59±16.63 | -9.85 | 355.55±21.79 | -11.11 | 363.88±5.13 | -9.03 | 373.16±9.99 | -6.71 |
| Psoralidin | 2.5 | 2.40±0.05 | -4.00 | 2.40±0.04 | -3.88 | 2.35±0.05 | -6.13 | 2.42±0.04 | -3.03 |
|  | 125 | 117.92±1.43 | -5.67 | 119.76±3.28 | -4.19 | 114.56±6.39 | -8.35 | 113.40±5.38 | -9.28 |
|  | 200 | 183.53±4.02 | -8.24 | 190.71±7.72 | -4.65 | 182.61±10.75 | -8.70 | 179.36±11.47 | -10.32 |
| Neobavaisoflavone | 1.25 | 1.19±0.05 | -5.07 | 1.24±0.04 | -1.16 | 1.21±0.03 | -3.17 | 1.21±0.02 | -2.98 |
|  | 25 | 23.08±1.17 | -7.67 | 23.03±1.45 | -7.90 | 22.88±1.54 | -8.48 | 23.67±1.07 | -5.34 |
|  | 40 | 36.42±4.13 | -8.95 | 34.72±2.67 | -13.20 | 36.22±2.97 | -9.46 | 34.82±2.38 | -12.95 |
| Bavachinin | 2.5 | 2.35±0.05 | -5.88 | 2.38±0.08 | -4.72 | 2.40±0.06 | -4.04 | 2.37±0.07 | -5.32 |
|  | 50 | 46.57±1.32 | -6.85 | 47.04±1.18 | -5.93 | 46.89±1.73 | -6.22 | 46.33±1.96 | -7.34 |
|  | 80 | 70.92±3.62 | -11.35 | 72.39±2.15 | -9.52 | 73.39±2.29 | -8.27 | 72.16±2.19 | -9.81 |

**Table 16. The stability of 12 compounds in rat liver (*n* = 6).**

| Compound | Concentrations  (ng/mL) | Freeze-thaw stability | | Short-term stability | | Long-term stability | | Pre-injection stability | |
| --- | --- | --- | --- | --- | --- | --- | --- | --- | --- |
|  |  | Measured  (ng/mL) | RE  (%) | Measured  (ng/mL) | RE  (%) | Measured  (ng/mL) | RE  (%) | Measured  (ng/mL) | RE  (%) |
| Psoralen | 12.5 | 12.38±0.09 | -0.98 | 12.44±0.03 | -0.51 | 12.43±0.04 | -0.53 | 12.44±0.04 | -0.52 |
|  | 500 | 490.75±4.55 | -1.85 | 487.43±6.73 | -2.51 | 489.28±7.15 | -2.14 | 486.88±9.53 | -2.62 |
|  | 800 | 779.93±11.47 | -2.51 | 762.21±17.79 | -4.72 | 771.31±26.44 | -3.59 | 764.97±21.26 | -4.38 |
| Isopsoralen | 12.5 | 12.43±0.05 | -0.54 | 12.44±0.02 | -0.46 | 12.46±0.02 | -0.33 | 12.44±0.04 | -0.50 |
|  | 500 | 488.25±7.40 | -2.35 | 490.76±6.57 | -1.85 | 487.61±6.29 | -2.48 | 482.72±6.79 | -3.46 |
|  | 800 | 774.93±13.94 | -3.13 | 765.38±17.34 | -4.33 | 772.65±25.02 | -3.42 | 764.97±20.77 | -4.38 |
| Psoralenoside | 12.5 | 12.46±0.01 | -0.30 | 12.45±0.04 | -0.43 | 12.46±0.02 | -0.36 | 12.43±0.03 | -0.53 |
|  | 500 | 490.59±4.73 | -1.88 | 491.43±2.74 | -1.71 | 485.94±7.01 | -2.81 | 482.72±8.13 | -3.46 |
|  | 800 | 774.92±15.55 | -3.13 | 767.05±21.14 | -4.12 | 773.81±27.83 | -3.27 | 764.97±21.26 | -4.38 |
| Bavachin | 2.5 | 2.46±0.01 | -1.74 | 2.43±0.04 | -2.75 | 2.44±0.05 | -2.56 | 2.42±0.07 | -3.12 |
|  | 250 | 242.25±4.24 | -3.10 | 241.93±4.62 | -3.23 | 239.28±8.83 | -4.29 | 238.55±7.24 | -4.58 |
|  | 400 | 379.93±20.02 | -5.02 | 368.88±12.25 | -7.78 | 376.31±18.38 | -5.92 | 379.97±18.53 | -5.01 |
| Isobavachin | 2.5 | 2.44±0.03 | -2.33 | 2.40±0.10 | -4.09 | 2.44±0.04 | -2.45 | 2.43±0.03 | -2.99 |
|  | 125 | 119.09±4.77 | -4.73 | 116.76±5.75 | -6.59 | 117.61±5.23 | -5.91 | 118.55±3.52 | -5.16 |
|  | 200 | 184.93±8.37 | -7.54 | 185.55±7.41 | -7.23 | 183.48±5.60 | -8.26 | 183.30±7.77 | -8.35 |
| Bavachalcone | 1.25 | 1.24±0.00 | -0.47 | 1.24±0.02 | -1.05 | 1.24±0.00 | -0.49 | 1.24±0.01 | -1.13 |
|  | 125 | 120.25±3.72 | -3.80 | 119.43±3.08 | -4.46 | 118.44±4.71 | -5.24 | 120.22±5.61 | -3.83 |
|  | 200 | 184.26±6.94 | -7.87 | 185.05±7.48 | -7.48 | 184.31±6.39 | -7.84 | 181.63±10.69 | -9.18 |
| Isobavachalcone | 2.5 | 2.46±0.01 | -1.50 | 2.45±0.04 | -2.16 | 2.46±0.02 | -1.78 | 2.43±0.03 | -2.65 |
|  | 50 | 48.09±1.56 | -3.83 | 47.26±3.33 | -5.48 | 47.61±2.44 | -4.78 | 46.88±3.29 | -6.23 |
|  | 80 | 76.59±2.83 | -4.26 | 74.55±3.22 | -6.82 | 75.81±3.93 | -5.23 | 72.97±4.42 | -8.79 |
| Pinoresinol diglucoside | 1.25 | 1.23±0.01 | -1.77 | 1.24±0.01 | -1.14 | 1.24±0.01 | -0.66 | 1.23±0.01 | -1.38 |
|  | 125 | 118.75±3.27 | -5.00 | 119.93±3.18 | -4.06 | 119.61±5.16 | -4.31 | 121.55±5.27 | -2.76 |
|  | 200 | 185.09±7.05 | -7.45 | 184.21±7.65 | -7.89 | 184.98±7.88 | -7.51 | 183.30±7.77 | -8.35 |
| Geniposidic acid | 2.5 | 2.46±0.01 | -1.50 | 2.45±0.04 | -2.16 | 2.46±0.01 | -1.71 | 2.43±0.02 | -2.71 |
|  | 500 | 482.75±16.06 | -3.45 | 471.09±32.77 | -5.78 | 476.94±23.40 | -4.61 | 474.38±22.83 | -5.12 |
|  | 800 | 765.59±28.95 | -4.30 | 743.71±32.24 | -7.04 | 757.48±38.23 | -5.31 | 730.80±45.30 | -8.65 |
| Psoralidin | 2.5 | 2.45±0.04 | -1.87 | 2.45±0.04 | -2.09 | 2.45±0.02 | -1.88 | 2.43±0.03 | -2.98 |
|  | 250 | 242.75±8.21 | -2.90 | 241.94±4.60 | -3.22 | 235.94±10.50 | -5.62 | 233.55±12.28 | -6.58 |
|  | 400 | 379.93±20.58 | -5.02 | 362.21±11.74 | -9.45 | 374.65±21.22 | -6.34 | 364.97±32.07 | -8.76 |
| Neobavaisoflavone | 2.5 | 2.46±0.02 | -1.57 | 2.45±0.04 | -2.03 | 2.46±0.02 | -1.45 | 2.45±0.04 | -1.85 |
|  | 500 | 483.59±17.84 | -3.28 | 472.93±32.24 | -5.41 | 477.94±23.16 | -4.41 | 479.38±20.49 | -4.12 |
|  | 800 | 766.76±29.41 | -4.16 | 744.55±31.05 | -6.93 | 755.81±40.58 | -5.52 | 737.47±46.13 | -7.82 |
| Bavachinin | 2.5 | 2.47±0.03 | -1.23 | 2.47±0.02 | -1.36 | 2.47±0.03 | -1.38 | 2.45±0.03 | -1.98 |
|  | 500 | 486.92±9.59 | -2.62 | 481.26±20.99 | -3.75 | 481.28±17.28 | -3.74 | 481.05±17.32 | -3.79 |
|  | 800 | 773.42±34.00 | -3.32 | 754.55±27.70 | -5.68 | 760.81±31.77 | -4.90 | 742.47±48.74 | -7.19 |

**Table 17. The stability of 12 compounds in rat spleen (*n* = 6).**

| Compound | Concentrations  (ng/mL) | Freeze-thaw stability | | Short-term stability | | Long-term stability | | Pre-injection stability | |
| --- | --- | --- | --- | --- | --- | --- | --- | --- | --- |
|  |  | Measured  (ng/mL) | RE  (%) | Measured  (ng/mL) | RE  (%) | Measured  (ng/mL) | RE  (%) | Measured  (ng/mL) | RE  (%) |
| Psoralen | 12.5 | 12.42±0.06 | -0.62 | 12.44±0.04 | -0.51 | 12.45±0.03 | -0.41 | 12.45±0.03 | -0.40 |
|  | 500 | 484.82±4.37 | -3.04 | 482.94±9.45 | -3.41 | 479.08±5.64 | -4.18 | 482.23±8.06 | -3.55 |
|  | 800 | 778.34±15.15 | -2.71 | 770.48±20.55 | -3.69 | 770.34±24.51 | -3.71 | 763.26±17.51 | -4.59 |
| Isopsoralen | 12.5 | 12.46±0.03 | -0.33 | 12.44±0.04 | -0.47 | 12.45±0.03 | -0.43 | 12.44±0.03 | -0.44 |
|  | 500 | 481.49±9.96 | -3.70 | 481.27±9.55 | -3.75 | 474.08±7.72 | -5.18 | 480.56±9.84 | -3.89 |
|  | 800 | 778.34±20.27 | -2.71 | 770.81±20.38 | -3.65 | 768.67±24.17 | -3.92 | 764.92±22.85 | -4.38 |
| Psoralenoside | 12.5 | 12.41±0.06 | -0.70 | 12.44±0.03 | -0.45 | 12.44±0.03 | -0.45 | 12.44±0.03 | -0.44 |
|  | 500 | 483.16±9.92 | -3.37 | 486.27±8.35 | -2.75 | 477.41±7.45 | -4.52 | 480.56±7.20 | -3.89 |
|  | 800 | 778.34±11.32 | -2.71 | 770.48±22.54 | -3.69 | 768.67±24.17 | -3.92 | 761.59±18.87 | -4.80 |
| Bavachin | 2.5 | 2.41±0.06 | -3.51 | 2.44±0.03 | -2.24 | 2.44±0.03 | -2.26 | 2.44±0.03 | -2.21 |
|  | 100 | 91.49±5.23 | -8.51 | 91.61±6.59 | -8.39 | 90.74±4.54 | -9.26 | 91.39±2.93 | -8.61 |
|  | 160 | 148.34±8.01 | -7.29 | 150.48±7.37 | -5.95 | 153.67±5.98 | -3.95 | 153.26±4.86 | -4.22 |
| Isobavachin | 1.25 | 1.24±0.01 | -0.70 | 1.24±0.01 | -0.89 | 1.23±0.01 | -1.92 | 1.22±0.01 | -2.31 |
|  | 50 | 47.16±1.68 | -5.69 | 46.61±2.62 | -6.79 | 44.08±2.02 | -11.85 | 45.06±3.58 | -9.88 |
|  | 80 | 75.01±2.83 | -6.24 | 73.81±3.56 | -7.73 | 77.01±2.37 | -3.74 | 71.76±5.89 | -10.31 |
| Bavachalcone | 1.25 | 1.23±0.02 | -1.65 | 1.22±0.02 | -2.03 | 1.23±0.01 | -1.56 | 1.23±0.01 | -1.78 |
|  | 25 | 23.32±1.19 | -6.70 | 23.27±1.03 | -6.91 | 22.74±1.09 | -9.03 | 22.89±1.33 | -8.42 |
|  | 40 | 36.01±2.31 | -9.97 | 36.65±2.20 | -8.38 | 37.01±2.37 | -7.48 | 36.92±2.03 | -7.70 |
| Isobavachalcone | 1.25 | 1.24±0.01 | -1.10 | 1.23±0.01 | -1.21 | 1.23±0.01 | -1.41 | 1.22±0.01 | -2.07 |
|  | 125 | 120.49±2.72 | -3.61 | 119.27±2.09 | -4.58 | 120.74±3.62 | -3.41 | 119.73±3.05 | -4.22 |
|  | 200 | 188.34±3.44 | -5.83 | 185.48±4.18 | -7.26 | 182.01±6.12 | -9.00 | 189.92±6.49 | -5.04 |
| Pinoresinol diglucoside | 1.25 | 1.23±0.02 | -1.90 | 1.23±0.01 | -1.42 | 1.23±0.01 | -1.85 | 1.23±0.01 | -1.94 |
|  | 50 | 47.32±1.90 | -5.36 | 47.27±2.33 | -5.46 | 46.58±2.48 | -6.85 | 46.56±2.97 | -6.88 |
|  | 80 | 75.84±2.49 | -5.20 | 75.48±3.22 | -5.65 | 72.01±4.69 | -9.99 | 73.92±4.92 | -7.60 |
| Geniposidic acid | 2.5 | 2.38±0.08 | -4.99 | 2.42±0.01 | -3.35 | 2.39±0.04 | -4.23 | 2.42±0.02 | -3.30 |
|  | 250 | 247.32±1.90 | -1.07 | 242.27±4.57 | -3.09 | 238.91±9.51 | -4.44 | 241.56±7.64 | -3.38 |
|  | 400 | 380.84±7.81 | -4.79 | 383.81±9.16 | -4.05 | 358.51±10.16 | -10.37 | 370.59±11.01 | -7.35 |
| Psoralidin | 2.5 | 2.36±0.07 | -5.66 | 2.41±0.02 | -3.58 | 2.41±0.02 | -3.69 | 2.42±0.02 | -3.06 |
|  | 250 | 243.99±6.53 | -2.41 | 238.94±7.67 | -4.42 | 233.91±11.62 | -6.44 | 244.89±8.26 | -2.04 |
|  | 400 | 370.84±13.23 | -7.29 | 385.48±11.59 | -3.63 | 371.84±11.89 | -7.04 | 373.92±12.01 | -6.52 |
| Neobavaisoflavone | 2.5 | 2.36±0.07 | -5.72 | 2.42±0.03 | -3.35 | 2.41±0.04 | -3.43 | 2.43±0.03 | -2.64 |
|  | 250 | 240.65±5.14 | -3.74 | 242.27±6.42 | -3.09 | 238.91±9.51 | -4.44 | 243.23±10.37 | -2.71 |
|  | 400 | 369.18±12.97 | -7.71 | 388.81±12.23 | -2.80 | 368.51±12.15 | -7.87 | 377.26±14.29 | -5.69 |
| Bavachinin | 2.5 | 2.39±0.04 | -4.39 | 2.43±0.04 | -2.69 | 2.41±0.04 | -3.48 | 2.44±0.02 | -2.33 |
|  | 500 | 480.65±7.65 | -3.87 | 452.27±12.97 | -9.55 | 477.24±8.20 | -4.55 | 479.89±12.92 | -4.02 |
|  | 800 | 772.51±12.45 | -3.44 | 780.48±15.78 | -2.44 | 773.51±11.37 | -3.31 | 767.26±25.33 | -4.09 |

**Table 18. The stability of 12 compounds in rat lung (*n* = 6).**

| Compound | Concentrations  (ng/mL) | Freeze-thaw stability | | Short-term stability | | Long-term stability | | Pre-injection stability | |
| --- | --- | --- | --- | --- | --- | --- | --- | --- | --- |
|  |  | Measured  (ng/mL) | RE  (%) | Measured  (ng/mL) | RE  (%) | Measured  (ng/mL) | RE  (%) | Measured  (ng/mL) | RE  (%) |
| Psoralen | 12.5 | 12.01±0.47 | -3.94 | 11.59±0.48 | -7.29 | 11.75±0.43 | -6.04 | 11.94±0.41 | -4.51 |
|  | 500 | 481.08±9.07 | -3.78 | 479.87±7.88 | -4.03 | 482.72±11.58 | -3.46 | 478.83±12.62 | -4.23 |
|  | 800 | 771.83±20.65 | -3.52 | 765.68±22.35 | -4.29 | 767.07±19.72 | -4.12 | 795.02±17.94 | -0.62 |
| Isopsoralen | 12.5 | 11.86±0.38 | -5.15 | 11.91±0.30 | -4.72 | 11.87±0.39 | -5.08 | 11.74±0.24 | -6.09 |
|  | 500 | 465.40±11.23 | -6.92 | 471.13±12.02 | -5.77 | 474.97±12.60 | -5.01 | 468.92±13.58 | -6.22 |
|  | 800 | 777.23±24.06 | -2.85 | 774.38±19.45 | -3.20 | 783.73±21.36 | -2.03 | 766.82±20.85 | -4.15 |
| Psoralenoside | 2.5 | 2.43±0.21 | -2.77 | 2.45±0.19 | -1.93 | 2.30±0.08 | -7.81 | 2.49±0.19 | -0.46 |
|  | 1000 | 990.00±39.92 | -1.00 | 983.33±24.09 | -1.67 | 988.67±36.25 | -1.13 | 970.83±28.78 | -2.92 |
|  | 1600 | 1516.83±49.42 | -5.20 | 1551.50±57.59 | -3.03 | 1454.50±73.76 | -9.09 | 1413.83±95.76 | -11.64 |
| Bavachin | 1.25 | 1.24±0.01 | -0.64 | 1.25±0.01 | -0.15 | 1.24±0.01 | -0.55 | 1.24±0.01 | -1.01 |
|  | 100 | 97.42±2.11 | -2.58 | 97.52±1.12 | -2.48 | 97.30±1.67 | -2.70 | 96.30±1.76 | -3.70 |
|  | 160 | 156.00±2.58 | 56.00 | 157.07±2.69 | -1.83 | 156.58±2.39 | -2.14 | 157.80±1.75 | -1.38 |
| Isobavachin | 1.25 | 1.24±0.01 | -0.09 | 1.24±0.01 | -0.52 | 1.25±0.02 | -0.14 | 1.24±0.01 | -0.63 |
|  | 50 | 49.20±1.03 | -1.60 | 49.63±1.78 | -0.74 | 48.35±1.30 | -3.31 | 49.02±1.41 | -1.97 |
|  | 80 | 77.65±2.94 | -2.94 | 78.15±1.14 | -2.31 | 78.97±1.72 | -1.29 | 78.65±1.42 | -1.69 |
| Bavachalcone | 1.25 | 1.22±0.03 | -2.19 | 1.24±0.01 | -0.98 | 1.24±0.01 | -0.61 | 1.24±0.01 | -0.60 |
|  | 50 | 49.47±0.86 | -1.07 | 49.48±0.82 | -1.04 | 48.06±0.77 | -3.88 | 48.75±0.92 | -2.50 |
|  | 80 | 78.68±1.12 | -1.65 | 78.70±1.75 | -1.63 | 78.32±1.00 | -2.10 | 78.58±1.32 | -1.78 |
| Isobavachalcone | 2.5 | 2.34±0.08 | -6.59 | 2.40±0.08 | -3.94 | 2.30±0.11 | -8.04 | 2.40±0.26 | -4.13 |
|  | 12.5 | 11.66±0.63 | -6.73 | 11.82±0.48 | -5.47 | 11.98±0.76 | -4.13 | 11.47±1.02 | -8.21 |
|  | 200 | 185.92±7.24 | -7.04 | 193.20±9.02 | -3.40 | 191.73±6.15 | -4.13 | 192.17±6.78 | -3.92 |
| Pinoresinol diglucoside | 1.25 | 1.25±0.01 | -0.26 | 1.25±0.01 | -0.34 | 1.24±0.01 | -0.42 | 1.25±0.01 | -0.35 |
|  | 250 | 247.60±1.49 | -0.96 | 245.95±3.61 | -1.62 | 248.42±1.46 | -0.63 | 248.13±3.52 | -0.75 |
|  | 400 | 375.28±8.27 | -6.18 | 392.72±9.61 | -1.82 | 374.33±8.35 | -6.42 | 391.97±8.61 | -2.01 |
| Geniposidic acid | 2.5 | 2.46±0.18 | -1.65 | 2.37±0.07 | -5.31 | 2.29±0.14 | -8.57 | 2.49±0.15 | -0.47 |
|  | 500 | 493.85±17.23 | -1.23 | 495.18±26.08 | -0.96 | 481.62±16.72 | -3.68 | 497.10±25.93 | -0.58 |
|  | 800 | 775.33±36.04 | -3.08 | 754.00±29.20 | -5.75 | 757.83±30.06 | -5.27 | 762.33±42.68 | -4.71 |
| Psoralidin | 2.5 | 2.37±0.19 | -5.28 | 2.34±0.13 | -6.31 | 2.31±0.11 | -7.59 | 2.33±0.07 | -6.63 |
|  | 250 | 244.38±3.68 | -2.25 | 242.28±7.61 | -3.09 | 244.83±8.55 | -2.07 | 238.91±2.87 | -4.44 |
|  | 400 | 393.63±12.36 | -1.59 | 388.25±17.74 | -2.94 | 385.06±12.04 | -3.73 | 381.64±10.69 | -4.59 |
| Neobavaisoflavone | 2.5 | 2.37±0.12 | -5.35 | 2.45±0.07 | -1.95 | 2.39±0.15 | -4.33 | 2.38±0.18 | -4.72 |
|  | 150 | 146.45±3.01 | -2.37 | 140.35±6.19 | -6.43 | 144.60±2.96 | -3.60 | 146.05±2.91 | -2.63 |
|  | 240 | 228.17±6.03 | -4.93 | 247.67±10.95 | 3.19 | 213.12±6.29 | -11.20 | 224.00±15.67 | -6.67 |
| Bavachinin | 2.5 | 2.48±0.07 | -0.95 | 2.43±0.04 | -2.87 | 2.43±0.03 | -2.87 | 2.47±0.03 | -1.28 |
|  | 250 | 248.92±2.49 | -0.43 | 244.87±2.90 | -2.05 | 246.55±2.79 | -1.38 | 245.50±2.65 | -1.80 |
|  | 400 | 388.50±9.99 | -2.88 | 380.68±9.64 | -4.83 | 387.07±5.81 | -3.23 | 390.02±6.70 | -2.50 |

**Table 19. The stability of 12 compounds in rat uterus (*n* = 6).**

| Compound | Concentrations  (ng/mL) | Freeze-thaw stability | | Short-term stability | | Long-term stability | | Pre-injection stability | |
| --- | --- | --- | --- | --- | --- | --- | --- | --- | --- |
|  |  | Measured  (ng/mL) | RE  (%) | Measured  (ng/mL) | RE  (%) | Measured  (ng/mL) | RE  (%) | Measured  (ng/mL) | RE  (%) |
| Psoralen | 2.5 | 2.30±0.19 | -8.03 | 2.44±0.13 | -2.53 | 2.34±0.07 | -6.31 | 2.48±0.11 | -0.84 |
|  | 500 | 492.35±17.55 | -1.53 | 493.48±15.31 | -1.30 | 495.98±16.14 | -0.80 | 482.47±17.36 | -3.51 |
|  | 800 | 790.78±31.55 | -1.15 | 794.10±27.22 | -0.74 | 789.32±26.03 | -1.33 | 791.16±32.05 | -1.11 |
| Isopsoralen | 2.5 | 2.45±0.06 | -1.88 | 2.45±0.06 | -1.87 | 2.46±0.05 | -1.53 | 2.47±0.05 | -1.2 |
|  | 250 | 244.07±7.10 | -2.37 | 245.05±5.05 | -1.98 | 244.70±8.42 | -2.12 | 245.36±7.06 | -1.86 |
|  | 400 | 382.76±14.05 | -4.31 | 388.47±12.20 | -2.88 | 389.72±8.53 | -2.57 | 391.32±19.96 | -2.17 |
| Psoralenoside | 12.5 | 12.34±0.20 | -1.30 | 12.39±0.24 | -0.87 | 12.39±0.24 | -0.87 | 12.41±0.09 | -0.69 |
|  | 1000 | 968.07±40.12 | -3.19 | 966.95±38.15 | -3.31 | 950.45±43.89 | -4.95 | 961.73±43.56 | -3.83 |
|  | 1600 | 1557.88±56.90 | -2.63 | 1551.84±46.30 | -3.01 | 1550.97±44.20 | -3.06 | 1566.09±48.50 | -2.12 |
| Bavachin | 2.5 | 2.43±0.05 | -2.83 | 2.44±0.06 | -2.56 | 2.40±0.08 | -4.03 | 2.42±0.07 | -3.29 |
|  | 100 | 97.68±4.08 | -2.32 | 99.03±4.55 | -0.97 | 95.38±5.13 | -4.62 | 100.05±5.22 | 0.05 |
|  | 160 | 159.28±10.32 | -0.45 | 161.47±13.50 | 0.92 | 161.52±10.19 | 0.95 | 157.17±12.08 | -1.77 |
| Isobavachin | 0.25 | 0.24±0.01 | -2.67 | 0.24±0.01 | -3.33 | 0.25±0.01 | -2 | 0.24±0.01 | -2.67 |
|  | 25 | 24.65±1.23 | -1.41 | 24.60±1.04 | -1.6 | 24.93±1.28 | -0.27 | 24.63±1.04 | -1.47 |
|  | 40 | 38.98±1.80 | -2.56 | 39.78±1.84 | -0.541 | 39.96±1.87 | -0.11 | 39.68±1.83 | -0.80 |
| Bavachalcone | 1.25 | 1.24±0.01 | -0.53 | 1.24±0.03 | -1.07 | 1.24±0.01 | -0.53 | 1.24±0.01 | -0.53 |
|  | 125 | 124.45±1.64 | -0.44 | 124.79±1.28 | -0.17 | 124.24±1.58 | -0.61 | 124.66±3.13 | -0.276 |
|  | 200 | 198.50±2.29 | -0.75 | 198.62±2.97 | -0.69 | 199.09±3.03 | -0.46 | 199.07±4.18 | -0.465 |
| Isobavachalcone | 2.5 | 2.48±0.03 | -0.93 | 2.48±0.04 | -0.8 | 2.49±0.03 | -0.47 | 2.49±0.07 | -0.27 |
|  | 25 | 24.40±1.28 | -2.42 | 24.93±0.42 | -0.27 | 24.61±1.18 | -1.56 | 24.72±0.84 | -1.11 |
|  | 40 | 39.24±3.12 | -1.90 | 40.22±1.71 | 0.56 | 39.20±2.12 | -1.20 | 39.69±2.12 | -0.77 |
| Pinoresinol diglucoside | 1.25 | 1.24±0.01 | -0.53 | 1.24±0.01 | -0.53 | 1.24±0.01 | -0.8 | 1.24±0.01 | -0.67 |
|  | 50 | 49.62±1.56 | -0.77 | 49.24±1.37 | -1.52 | 49.50±1.95 | -1 | 49.66±1.43 | -0.68 |
|  | 80 | 79.30±2.61 | -0.86 | 78.65±2.76 | -1.69 | 79.82±3.13 | -0.23 | 79.29±2.96 | -0.89 |
| Geniposidic acid | 1.25 | 1.24±0.01 | -0.53 | 1.24±0.01 | -0.67 | 1.24±0.01 | -0.53 | 1.24±0.01 | -0.67 |
|  | 250 | 247.87±8.47 | -0.85 | 243.73±2.58 | -2.51 | 242.80±4.51 | -2.88 | 245.60±5.57 | -1.76 |
|  | 400 | 390.77±12.50 | -2.31 | 378.92±13.50 | -5.27 | 382.61±12.05 | -4.35 | 385.60±12.49 | -3.6 |
| Psoralidin | 2.5 | 2.42±0.15 | -3.21 | 2.46±0.16 | -1.49 | 2.46±0.10 | -1.64 | 2.47±0.05 | -1.2 |
|  | 125 | 124.93±2.97 | -0.056 | 123.99±2.25 | -0.81 | 123.97±1.76 | -0.82 | 123.93±2.58 | -0.86 |
|  | 200 | 199.36±4.38 | -0.32 | 199.78±8.14 | -0.11 | 200.45±5.74 | 0.23 | 199.47±6.88 | -0.27 |
| Neobavaisoflavone | 2.5 | 2.49±0.02 | -0.4 | 2.48±0.03 | -0.87 | 2.49±0.08 | -0.32 | 2.49±0.10 | -0.6 |
|  | 25 | 23.97±0.95 | -4.11 | 24.09±0.76 | -3.64 | 24.31±0.65 | -2.75 | 24.06±0.81 | -3.75 |
|  | 40 | 35.60±2.12 | -11.00 | 37.85±1.07 | -5.37 | 39.01±1.86 | -2.48 | 38.98±1.61 | -2.55 |
| Bavachinin | 2.5 | 2.49±0.03 | -0.6 | 2.48±0.04 | -0.93 | 2.50±0.04 | -0.2 | 2.47±0.05 | -1.2 |
|  | 25 | 24.60±1.12 | -1.6 | 24.65±1.04 | -1.4 | 24.95±1.39 | -0.2 | 24.27±1.05 | -2.93 |
|  | 40 | 39.72±1.71 | -0.71 | 39.33±1.96 | -1.68 | 38.90±1.93 | -2.75 | 39.84±2.10 | -0.41 |

**Table 20. The stability of 12 compounds in rat ovary (*n* = 6).**

| Compound | Concentrations  (ng/mL) | Freeze-thaw stability | | | Short-term stability | | | Long-term stability | | | Pre-injection stability | | |
| --- | --- | --- | --- | --- | --- | --- | --- | --- | --- | --- | --- | --- | --- |
|  |  | Measured  (ng/mL) | | RE  (%) | Measured  (ng/mL) | RE  (%) | | Measured  (ng/mL) | | RE  (%) | Measured  (ng/mL) | RE  (%) | |
| Psoralen | 12.5 | 12.32±0.43 | -1.48 | | 12.40±0.58 | | -0.8 | 12.38±0.47 | -1 | | 12.41±0.55 | | -0.72 |
|  | 500 | 492.67±39.53 | -1.47 | | 494.23±34.28 | | -1.15 | 484.52±35.10 | -3.10 | | 489.40±40.54 | | -2.12 |
|  | 800 | 789.08±55.74 | -1.36 | | 776.65±57.01 | | -2.92 | 761.57±55.30 | -4.80 | | 762.82±57.41 | | -4.65 |
| Isopsoralen | 12.5 | 12.18±0.35 | -2.56 | | 12.38±0.28 | | -0.93 | 12.46±0.34 | -0.33 | | 12.46±0.42 | | -0.32 |
|  | 500 | 489.97±45.79 | -2.01 | | 486.17±42.32 | | -2.77 | 489.05±38.29 | -2.19 | | 485.82±42.84 | | -2.84 |
|  | 800 | 785.40±59.95 | -1.825 | | 785.50±64.21 | | -1.81 | 762.83±59.97 | -4.65 | | 772.00±65.64 | | -3.5 |
| Psoralenoside | 12.5 | 12.46±0.04 | -0.33 | | 12.40±0.30 | | -0.81 | 12.42±0.09 | -0.67 | | 12.35±0.39 | | -1.21 |
|  | 1000 | 998.00±52.84 | -0.2 | | 994.00±51.27 | | -0.6 | 980.67±55.36 | -1.93 | | 1000.33±59.79 | | 0.03 |
|  | 1600 | 1585.00±79.70 | -0.94 | | 1580.67±72.63 | | -1.21 | 1573.33±72.98 | -1.67 | | 1576.17±92.14 | | -1.49 |
| Bavachin | 2.5 | 2.44±0.06 | -2.33 | | 2.41±0.08 | | -3.67 | 2.44±0.07 | -2.4 | | 2.47±0.05 | | -1.07 |
|  | 25 | 24.47±1.42 | -2.13 | | 24.78±1.40 | | -0.87 | 24.78±1.46 | -0.87 | | 24.82±1.21 | | -0.73 |
|  | 40 | 38.88±2.23 | -2.79 | | 39.80±2.41 | | -0.5 | 39.18±2.43 | -2.04 | | 39.13±2.08 | | -2.17 |
| Isobavachin | 2.5 | 2.48±0.05 | -0.84 | | 2.44±0.05 | | -2.35 | 2.42±0.08 | -3.33 | | 2.32±0.09 | | -7.24 |
|  | 25 | 24.45±1.32 | -2.21 | | 24.33±1.42 | | -2.67 | 24.67±1.33 | -1.33 | | 24.51±1.53 | | -1.98 |
|  | 40 | 39.70±2.39 | -0.75 | | 36.36±3.15 | | -9.10 | 38.28±2.99 | -4.30 | | 39.03±2.19 | | -2.42 |
| Bavachalcone | 1.25 | 1.24±0.01 | -1.07 | | 1.25±0.02 | | -0.4 | 1.24±0.03 | -0.8 | | 1.23±0.01 | | -1.73 |
|  | 25 | 24.81±1.62 | -0.76 | | 24.20±2.20 | | -3.22 | 24.70±1.88 | -1.19 | | 24.75±2.07 | | -1 |
|  | 40 | 38.87±4.41 | -2.82 | | 39.15±4.00 | | -2.13 | 38.34±3.54 | -4.16 | | 38.97±4.07 | | -2.58 |
| Isobavachalcone | 1.25 | 1.24±0.02 | -0.8 | | 1.25±0.03 | | -0.387 | 1.25±0.05 | 0.13 | | 1.24±0.02 | | -0.53 |
|  | 25 | 24.85±1.51 | -0.62 | | 24.83±2.03 | | -0.67 | 24.82±1.60 | -0.72 | | 24.81±1.82 | | -0.77 |
|  | 40 | 39.72±4.92 | -0.70 | | 39.39±3.83 | | -1.52 | 38.73±3.51 | -3.18 | | 38.92±3.72 | | -2.71 |
| Pinoresinol diglucoside | 1.25 | 1.25±0.04 | -0.4 | | 1.24±0.03 | | -0.8 | 1.23±0.02 | -1.33 | | 1.24±0.03 | | -1.09 |
|  | 50 | 49.68±2.02 | -0.63 | | 49.98±2.20 | | -0.03 | 49.03±2.25 | -1.93 | | 48.82±1.85 | | -2.37 |
|  | 80 | 79.02±3.47 | -1.23 | | 78.80±3.30 | | -1.5 | 79.58±3.15 | -0.52 | | 78.05±3.04 | | -2.44 |
| Geniposidic acid | 2.5 | 2.51±0.21 | 0.3 | | 2.44±0.09 | | -2.49 | 2.44±0.07 | -2.53 | | 2.46±0.06 | | -1.8 |
|  | 500 | 470.92±21.53 | -5.82 | | 481.03±26.59 | | -3.79 | 488.60±23.55 | -2.28 | | 491.87±25.79 | | -1.63 |
|  | 800 | 759.52±44.49 | -5.06 | | 771.65±42.90 | | -3.54 | 780.98±40.08 | -2.38 | | 777.50±38.24 | | -2.81 |
| Psoralidin | 1.25 | 1.24±0.03 | -0.8 | | 1.24±0.04 | | -0.92 | 1.23±0.03 | -1.33 | | 1.23±0.04 | | -1.87 |
|  | 50 | 48.99±2.15 | -2.03 | | 49.88±2.86 | | -0.24 | 49.83±2.63 | -0.35 | | 49.48±2.49 | | -1.03 |
|  | 80 | 79.61±4.44 | -0.49 | | 78.45±4.26 | | -1.93 | 78.95±4.56 | -1.31 | | 77.81±4.88 | | -2.73 |
| Neobavaisoflavone | 2.5 | 2.47±0.10 | -1.07 | | 2.35±0.10 | | -6.06 | 2.37±0.10 | -5.39 | | 2.48±0.09 | | -0.69 |
|  | 50 | 49.07±2.01 | -1.85 | | 49.90±2.55 | | -0.2 | 49.49±2.75 | -1.02 | | 49.09±2.23 | | -1.82 |
|  | 80 | 79.48±5.38 | -0.65 | | 78.83±4.77 | | -1.46 | 78.67±4.19 | -1.67 | | 78.60±4.41 | | -1.75 |
| Bavachinin | 2.5 | 2.41±0.06 | -3.47 | | 2.42±0.08 | | -3.4 | 2.44±0.06 | -2.4 | | 2.45±0.05 | | -1.93 |
|  | 25 | 24.50±1.23 | -2 | | 24.63±1.51 | | -1.5 | 24.63±1.61 | -1.47 | | 24.82±1.55 | | -0.73 |
|  | 40 | 39.14±2.40 | -2.14 | | 39.42±2.75 | | -1.46 | 38.97±2.67 | -2.58 | | 38.68±2.05 | | -3.3 |

**Table 21. The stability of 12 compounds in rat testes (*n* = 6).**

| Compound | Concentrations  (ng/mL) | Freeze-thaw stability | | Short-term stability | | Long-term stability | | Pre-injection stability | |
| --- | --- | --- | --- | --- | --- | --- | --- | --- | --- |
|  |  | Measured  (ng/mL) | RE  (%) | Measured  (ng/mL) | RE  (%) | Measured  (ng/mL) | RE  (%) | Measured  (ng/mL) | RE  (%) |
| Psoralen | 2.5 | 2.40±0.08 | -3.98 | 2.38±0.11 | -4.98 | 2.40±0.07 | -4.14 | 2.36±0.09 | -5.51 |
|  | 500 | 479.92±30.31 | -4.02 | 481.78±15.37 | -3.64 | 475.52±19.43 | -4.90 | 469.78±28.05 | -6.04 |
|  | 800 | 762.31±30.66 | -4.71 | 770.68±30.56 | -3.66 | 759.90±30.29 | -5.01 | 761.95±35.31 | -4.76 |
| Isopsoralen | 12.5 | 12.06±0.28 | -3.56 | 12.38±0.16 | -0.98 | 12.36±0.13 | -1.12 | 12.39±0.10 | -0.88 |
|  | 500 | 495.25±8.83 | -0.95 | 496.83±10.48 | -0.63 | 495.38±5.13 | -0.92 | 495.06±6.69 | -0.99 |
|  | 800 | 767.97±13.72 | -4.00 | 787.02±14.78 | -1.62 | 750.95±28.16 | -6.13 | 774.65±25.95 | -3.17 |
| Psoralenoside | 12.5 | 12.16±0.07 | -2.69 | 11.90±0.52 | -4.77 | 11.95±1.06 | -4.39 | 12.22±0.41 | -2.24 |
|  | 500 | 485.38±22.30 | -2.92 | 478 .92±26.98 | -4.22 | 470.47±39.54 | -5.91 | 487.59±7.94 | -2.48 |
|  | 800 | 752.47±24.06 | -5.94 | 748.73±38.03 | -6.41 | 748.38±26.48 | -6.45 | 733.92±29.16 | -8.26 |
| Bavachin | 2.5 | 2.28±0.14 | -8.73 | 2.32±0.16 | -7.37 | 2.36±0.11 | -5.57 | 2.35±0.07 | -6.19 |
|  | 25 | 23.58±1.49 | -5.70 | 23.07±1.05 | -7.74 | 23.52±1.38 | -5.90 | 23.10±1.59 | -7.61 |
|  | 40 | 37.00±1.81 | -7.50 | 36.81±1.59 | -7.98 | 37.49±1.48 | -6.28 | 36.74±1.91 | -8.16 |
| Isobavachin | 1.25 | 1.19±0.02 | -4.57 | 1.20±0.04 | -4.02 | 1.21±0.04 | -2.81 | 1.20±0.04 | -3.64 |
|  | 25 | 23.84±0.82 | -4.66 | 23.54±0.92 | -5.85 | 24.02±0.90 | -3.91 | 23.71±1.06 | -5.17 |
|  | 40 | 37.72±1.36 | -5.70 | 37.42±2.41 | -6.45 | 38.19±1.16 | -4.53 | 36.50±3.67 | -8.76 |
| Bavachalcone | 1.25 | 1.22±0.03 | -2.58 | 1.20±0.04 | -4.10 | 1.21±0.05 | -3.35 | 1.19±0.03 | -4.48 |
|  | 25 | 24.02±0.75 | -3.92 | 23.46±1.41 | -6.17 | 23.86±1.29 | -4.57 | 23.55±1.12 | -5.81 |
|  | 40 | 38.01±1.93 | -4.98 | 36.95±1.84 | -7.63 | 37.96±1.92 | -5.10 | 37.43±1.74 | -6.43 |
| Isobavachalcone | 1.25 | 1.20±0.02 | -4.38 | 1.22±0.03 | -2.71 | 1.20±0.03 | -4.18 | 1.21±0.05 | -2.92 |
|  | 25 | 23.83±1.25 | -4.67 | 24.21±1.41 | -3.17 | 23.88±1.86 | -4.50 | 24.19±1.66 | -3.23 |
|  | 40 | 37.28±1.59 | -6.80 | 38.11±1.86 | -4.72 | 37.68±2.77 | -5.80 | 37.92±2.56 | -5.19 |
| Pinoresinol diglucoside | 1.25 | 1.19±0.06 | -4.50 | 1.19±0.06 | -4.74 | 1.19±0.04 | -4.90 | 1.19±0.05 | -4.79 |
|  | 25 | 23.71±1.21 | -5.18 | 23.63±0.63 | -5.48 | 23.75±1.29 | -5.00 | 23.70±3.14 | -5.20 |
|  | 40 | 37.47±2.83 | -6.33 | 37.69±3.10 | -5.78 | 37.31±2.67 | -6.73 | 37.58±3.40 | -6.05 |
| Geniposidic acid | 2.5 | 2.39±0.07 | -4.41 | 2.40±0.12 | -4.13 | 2.38±0.13 | -4.89 | 2.40±0.10 | -4.09 |
|  | 125 | 117.22±6.69 | -6.23 | 117.25±8.97 | -6.20 | 116.80±6.14 | -6.56 | 119.59±7.12 | -4.33 |
|  | 200 | 182.47±10.07 | -8.77 | 185.07±8.44 | -7.47 | 184.22±15.34 | -7.89 | 182.75±11.89 | -8.63 |
| Psoralidin | 1.25 | 1.18±0.05 | -5.54 | 1.19±0.05 | -4.88 | 1.18±0.23 | -5.94 | 1.19±0.04 | -4.81 |
|  | 25 | 23.45±0.71 | -6.21 | 22.99±1.74 | -8.04 | 23.20±1.15 | -7.21 | 23.44±1.59 | -6.25 |
|  | 40 | 37.46±0.92 | -6.36 | 36.63±2.39 | -8.44 | 36.11±1.38 | -9.73 | 36.98±2.99 | -7.55 |
| Neobavaisoflavone | 2.5 | 2.41±0.05 | -3.57 | 2.44±0.06 | -2.21 | 2.42±0.12 | -3.15 | 2.41±0.06 | -3.53 |
|  | 25 | 24.06±0.78 | -3.78 | 24.06±1.55 | -3.75 | 23.40±1.10 | -6.41 | 24.04±2.12 | -3.84 |
|  | 40 | 36.82±3.46 | -7.95 | 37.91±4.27 | -5.24 | 36.89±4.83 | -7.77 | 37.35±3.12 | -6.62 |
| Bavachinin | 2.5 | 2.37±0.06 | -5.32 | 2.37±0.06 | -5.03 | 2.39±0.05 | -4.45 | 2.39±0.14 | -4.41 |
|  | 50 | 48.58±1.40 | -2.83 | 46.40±1.04 | -7.21 | 48.77±2.35 | -2.47 | 47.06±3.03 | -5.87 |
|  | 80 | 74.97±2.15 | -6.29 | 75.85±4.02 | -5.19 | 78.45±1.87 | -1.94 | 76.32±3.00 | -4.60 |
